# HAMLET Binding to α-Actinin Facilitates Tumor Cell Detachment

**DOI:** 10.1371/journal.pone.0017179

**Published:** 2011-03-08

**Authors:** Maria Trulsson, Hao Yu, Lennart Gisselsson, Yinxia Chao, Alexander Urbano, Sonja Aits, Ann-Kristin Mossberg, Catharina Svanborg

**Affiliations:** 1 Institute of Laboratory Medicine, Department of Microbiology, Immunology and Glycobiology, Lund University, Lund, Sweden; 2 Phase Holographic Imaging AB, Lund, Sweden; 3 Singapore Immunology Network (SIgN), Agency for Science, Technology, and Research (A*STAR), Singapore, Singapore; Vanderbilt University Medical Center, United States of America

## Abstract

Cell adhesion is tightly regulated by specific molecular interactions and detachment from the extracellular matrix modifies proliferation and survival. HAMLET (Human Alpha-lactalbumin Made LEthal to Tumor cells) is a protein-lipid complex with tumoricidal activity that also triggers tumor cell detachment in vitro and in vivo, suggesting that molecular interactions defining detachment are perturbed in cancer cells. To identify such interactions, cell membrane extracts were used in Far-western blots and HAMLET was shown to bind α-actinins; major F-actin cross-linking proteins and focal adhesion constituents. Synthetic peptide mapping revealed that HAMLET binds to the N-terminal actin-binding domain as well as the integrin-binding domain of α-actinin-4. By co-immunoprecipitation of extracts from HAMLET-treated cancer cells, an interaction with α-actinin-1 and -4 was observed. Inhibition of α-actinin-1 and α-actinin-4 expression by siRNA transfection increased detachment, while α-actinin-4-GFP over-expression significantly delayed rounding up and detachment of tumor cells in response to HAMLET. In response to HAMLET, adherent tumor cells rounded up and detached, suggesting a loss of the actin cytoskeletal organization. These changes were accompanied by a reduction in β1 integrin staining and a decrease in FAK and ERK1/2 phosphorylation, consistent with a disruption of integrin-dependent cell adhesion signaling. Detachment per se did not increase cell death during the 22 hour experimental period, regardless of α-actinin-4 and α-actinin-1 expression levels but adherent cells with low α-actinin levels showed increased death in response to HAMLET. The results suggest that the interaction between HAMLET and α-actinins promotes tumor cell detachment. As α-actinins also associate with signaling molecules, cytoplasmic domains of transmembrane receptors and ion channels, additional α-actinin-dependent mechanisms are discussed.

## Introduction

Cell adhesion is essential for tissue integrity and processes that modify adhesion are tightly regulated [1]. In normal cells, disrupted adhesion attenuates nutrient and growth factor access and may activate cell death [1], [2]. Cancer cells, in contrast, are frequently able to grow in an anchorage-independent way and detachment from the site of primary tumor growth may constitute a first step in metastatic spread. The detachment process requires complex modifications of molecular interactions in specific intercellular adhesion complexes as well as with the extracellular matrix through focal adhesion complexes [3], with key components including integrins and proteins linking the integrins to the cytoskeleton such as α-actinin, talin, tensin, filamin, vinculin and paxillin. In addition, a wide range of proteins modifies cell adhesion complexes by controlling the structure or activation state of their constituents [4].

α-Actinin functions as a scaffold between actin filaments and β integrins. Actin binds to the N-terminal domains of the anti-parallel α-actinin homodimer and β integrins recognize the spectrin-like repeats [4], [5], [6], [7]. So far, four human α-actinin isoforms have been described. α-Actinin-1 is found in focal adhesions and various F-actin-based structures [8], [9], [10]. α-Actinin-2 and α-actinin-3 are expressed in cardiac and/or skeletal muscles and cross-link F-actin in the region of Z-discs of muscle cells [11]. α-Actinin-4, which displays 87% sequence identity to α-actinin-1 [9], is detected at points of cell-cell contact [12] and interacts with focal adhesion constituents, including vinculin and the cytoplasmic domain of β integrins [3], [6], [10]. α-Actinin has been proposed to play a crucial role in the step of de-adhesion e.g. by recruiting MEKK1 and calpains which cleave several focal adhesion proteins (reviewed in [13]). In addition, α-actinin-4 deficient cells detach more easily in response to shear stress than cells with physiological α-actinin-4 expression, further suggesting a role in cell adhesion [14]. Furthermore, it has been shown that inhibition of α-actinin-4 by shRNA decreased cell-matrix adhesion in some astrocytoma cell lines while α-actinin-1 silencing had no effect or even increased the adhesion [15]. In addition to their role as actin cross-linkers, α-actinins associate with signaling molecules, cytoplasmic domains of transmembrane receptors and ion channels, connecting the cytoskeletal scaffold to diverse signaling processes [16].

HAMLET is a complex consisting of partially unfolded α-lactalbumin and oleic acid, which kills tumor cells and immature cells but not normal differentiated cells [17]. Early *in vitro* experiments showed that HAMLET displays broad anti-tumor activity [18], [19]. Subsequent therapeutic studies have supported these findings and suggested that HAMLET retains its tumoricidal activity *in vivo*. Topical HAMLET administration reduced lesion size in patients with skin papillomas [20] and HAMLET treatment delayed tumor progression and increased survival in a rat glioblastoma xenograft model without evidence of cell death in healthy brain tissue [21]. In patients with bladder cancer, local HAMLET instillations killed tumor cells but not healthy cells in surrounding tissues. During the bladder cancer study, we observed that HAMLET triggered massive shedding of dead tumor cells into the urine, suggesting that HAMLET might perturb tumor cell adhesion *in vivo* by targeting cell adhesion molecules and their regulatory pathways [20].

In the present study, we have characterized the detachment process in greater detail. We identify α-actinin-1 and -4 as molecular targets for HAMLET and show that interference with α-actinin function promotes detachment. Tumor cell detachment in response to HAMLET is accompanied by disruption of cytoskeletal structure and reduced focal adhesion kinase (FAK) phosphorylation and MAPK/ERK kinase (MEK)/extracellular-signal regulated kinase 1/2 (ERK1/2) signaling. The modification by HAMLET of cell morphology and adherence was carcinoma cell-specific, as normal differentiated cells remained adherent and retained their morphology in the presence of HAMLET.

## Materials and Methods

### HAMLET production

HAMLET was produced as previously described [17]. Briefly, α-lactalbumin was purified from human milk by hydrophobic interaction chromatography. The protein was unfolded with EDTA and subjected to ion-exchange chromatography on a matrix pre-conditioned with oleic acid and eluted with high salt. HAMLET was lyophilized after purification and stored at -20°C until used.

### Cellular assays

Lung carcinoma cells (A549, ATCC, Manassas, VA) were cultured in RPMI-1640 with non-essential amino acids (1∶100), 1 mM sodium pyruvate (all from PAA, Pasching, Austria), 50 µg/ml Gentamicin (Gibco, Paisley, UK) and 5% fetal calf serum (FCS). Primary cultures of human renal tubular epithelial cells (HRTEC, [22]) were cultured in DMEM (F12, PAA) with 15% FCS. Cells (5*10^5^/ml for cells in suspension, 40000 cells/well for adherent cells) were incubated with HAMLET in serum-free RPMI-1640 at 37°C. FCS was added after 1 hour. Cell death was quantified by trypan blue exclusion (Chroma Gesellschaft Schmid & Co, Münster, Germany) or by ATP measurements (ATPlite Kit, PerkinElmer, Waltham, MA, LUMIstar Luminometer, BMG LABTECH, Offenburg, Germany or Infinite F200, Tecan Group Ltd., Männedorf, Switzerland).

### Blots and co-immunoprecipitation

Proteins were extracted from A549 cells, separated with the Qproteome Cell Compartment kit (QIAGEN, Hilden, Germany) into cytosolic, nuclear and cytoskeletal fractions and a fraction with membrane and membrane-enclosed organelles. Membrane fractions (10 µg) were subjected to SDS-PAGE on 4–12% Bis-Tris gels (Invitrogen, Carlsbad, CA). One gel was stained with Coomassie and a parallel gel was blotted onto PVDF membranes. After saturation of the membranes (Sat-1 and Sat-2, [23]), the blots were incubated overnight with HAMLET dissolved in NN8 buffer (5 g/l gelatine hydrolysate, 1 g/l Tween-20, 20.5 g/l NaCl, 0.24 g/l Tris Base, pH 8.7) to 142 µM. Bound HAMLET was detected using anti-bovine α-lactalbumin antibodies (1∶16000, Bethyl Laboratories, Montgomery, TX), with horseradish peroxidase-conjugated donkey anti-goat secondary antibodies (1∶5000, Santa Cruz Biotechnology, Santa Cruz, CA), ECL Plus Western Blotting Reagent (GE Healthcare, Little Chalfont, UK) and GelDoc equipment (Bio-Rad Laboratories, Hercules, CA). After identification of the HAMLET-binding proteins, the corresponding bands were excised from the gel, digested and identified by MALDI-TOF mass spectrometry (Reflex™ III MALDI-TOF mass spectrometer (Bruker Daltonics, Bremen, Germany)) and a ProFound search (http://prowl.rockefeller.edu/, Rockefeller University).

For Western blots for siRNA experiments, the cells were detached and the same number of cells from each sample was lysed in NP-40 buffer (50 mM Tris-HCl, pH 7.8, 1% NP-40, 2 mM EDTA, 150 mM NaCl) containing Complete protease inhibitor cocktail (Roche Diagnostics, Mannheim, Germany). For FAK and ERK1/2 Western blots, adherent cells were washed with PBS containing 0.2 mM PMSF, 1 µg/ml Pepstatin A, 5 µg/ml Leupeptin (Sigma-Aldrich, St. Louis, MO) and Complete protease inhibitor cocktail. RIPA lysis buffer [24] containing the same protease inhibitors was added to the wells and the cells were removed from the plates using cell scrapers. As a control, some cells were detached with versen (EDTA) and lysed. The lysates where cleared by centrifugation and protein concentrations were measured using the DC Protein Assay (Bio-Rad Laboratories). Equal amounts of protein or, for siRNA experiments, equal volumes of lysates were separated by SDS-PAGE on 4-12% Bis-Tris gels (Invitrogen) and blotted onto PVDF membranes. Membranes were saturated with BSA (α-actinin-1 and -4, GAPDH) or nonfat dry milk (phospho-FAK, FAK, phospho-ERK1/2, ERK1/2, β1 integrin) and incubated with anti-α-actinin-4 (1∶2000, Alexis Biochemicals, San Diego, CA), anti-α-actinin-1 (1∶300, Abcam, Cambridge, UK; 1∶2000, Epitomics, Burlingame, CA), anti-FAK, anti-phospho-Y397-FAK (both 1∶1000, BD Biosciences, San Jose, CA), anti-p44/42, anti-phospho-(Thr202/Tyr204)-ERK1/2 (both 1∶1000, Cell Signaling Technology, Danvers, MA), anti-β1 integrin (1∶500, Epitomics) or anti-GAPDH (1∶3000–5000, Novus Biologicals, Littleton, CO) antibodies, followed by horseradish peroxidase-conjugated secondary anti-rabbit (1∶1000–2000, DakoCytomation, Glostrup, Denmark) or anti-mouse (1∶20000–50000, Novus Biologicals) antibodies. Bound antibodies were detected with ECL Plus Western Blotting Reagent (GE Healthcare) and GelDoc equipment (Bio-Rad Laboratories). If required, membranes were stripped with Restore Western Blot Stripping Buffer (Pierce, Rockford, IL), reblocked and reprobed with new antibodies.

For co-immunoprecipitation, HAMLET-treated cells were lysed in Brij 96 [25] (β1 integrin) or NP-40 lysis buffer (α-actinin-1 and -4) supplemented with Complete protease inhibitor cocktail. Lysates were incubated with rec-Protein G-Sepharose® 4B beads (Invitrogen) to reduce unspecific binding. Anti-α-lactalbumin antibodies (5-10 µg/ml, Bethyl Laboratories) or anti-α-actinin-4 antibodies (25 µg/ml, Santa Cruz Biotechnology, Santa Cruz, CA) were then added to half of the lysate, using the second half as a control and incubated with gentle agitation at 4°C. After 1 hour, fresh rec-Protein G-Sepharose® 4B beads were added and the incubation continued overnight. Beads with bound proteins were collected by centrifugation (12000×g, 30 sec) and washed three times in lysis buffer. The proteins were recovered by boiling the beads in LDS sample buffer (Invitrogen) with 100 mM DTT and used for Western blot.

### Peptide-binding assay

Maxi Sorp 96-well plates (NUNC A/S, Roskilde, Denmark) coated with HAMLET (2 µg/ml) were incubated with biotinylated α-actinin-4 peptides (200 ng/ml), 20 amino acids long with a 5 amino acid overlap (Innovagen, Lund, Sweden). Bound peptides were detected with streptavidin-alkaline phosphatase conjugate (1∶1000, Mabtech, Stockholm, Sweden) and phosphatase substrate (Sigma Aldrich) in 1M diethanolamine pH 9.8 at 405 nm. The PDB file of cryo-EM determined smooth muscle α-actinin from chicken gizzard (PDB code 1SJJ; [26]) was used for structural representation of the protein using MolMol [27].

### Immunostaining

For confocal microscopy, cells grown overnight on 8-well chamber slides (Nalge Nunc, Rochester, NY) were treated with HAMLET (10% was labeled with Alexa-Fluor 568 (Molecular Probes, Eugene, OR)) in serum-free medium and fixed in 3.7% formaldehyde. Detached cells where centrifuged onto microscope slides. All cells were permeabilized (0.25% Triton X-100/5% FCS/phosphate buffered saline, 10 minutes) and stained with anti-α-actinin-4 (1∶250, Alexis Biochemicals) or anti-β1 integrin (1∶100, Abcam) antibodies followed by Alexa-Fluor 488-labeled secondary anti-rabbit antibodies or Alexa-Fluor 568-labeled secondary anti-mouse antibodies (1∶100, Molecular Probes). Nuclei were stained using DRAQ5 (eBioscience, San Diego, CA). For the peptide inhibition studies, equimolar amounts of HAMLET and peptide were pre-incubated for 1 hour before being added to the cells. Images were captured on a LSM510 META confocal microscope (Carl Zeiss, Jena, Germany). Fluorescence was detected with pinhole settings corresponding to 1 airy unit.

For live cell imaging, nuclei were stained with Hoechst 33342 (Invitrogen) before HAMLET (21 µM) was added. The cells were kept at 37°C, 5% CO_2_ and examined unfixed in a LSM510 DUO confocal microscope (Carl Zeiss).

### Cell detachment

A549 cells were allowed to adhere overnight (40 000 cells/well in a 24-well plate). Increasing cell numbers were plated as a standard curve. The wells were washed with PBS and new medium without FCS was added together with HAMLET. For time points longer than 1 hour, FCS was added after 1 hour. The number of cells present in the well was quantified by measuring the hexosaminidase activity [28] in each well. Briefly, the medium was removed, the wells washed with PBS and the hexosaminidase substrate [28] was added. After 2 hours of incubation at 37°C, 60 µl sample was mixed with 90 µl STOP solution [28] and the absorbance was measured at 405 nm.

### Phase Holographic Imaging

The HoloMonitor™ M2 digital holographic microscope (Phase Holographic Imaging AB, Lund, Sweden) records 3D information of cells using interfering wave fronts induced by the exposure to a 0.8 mW HeNe laser (633 nm), resembling a Mach-Zender interferometer where the cells, placed in one of the interfering wave fronts, induce a phase difference between the two beams [29], [30], [31]. The interference pattern (hologram) is recorded on a digital sensor and is used to reconstruct the amplitude and phase of the object as described [32], [33]. Cells grown on µ-Slide I coated with ibiTreat (ibidi, Martinsried, Germany) were treated with HAMLET and images were captured with an imaging time of 2.4 msec.

### Transfections

A549 cells were transfected with α-actinin-4 siRNA (sense sequence GCAGCAUCGUGGACUACAATT), α-actinin-1 siRNA (sense sequence CACCAUGCAUGCCAUGCAA) or AllStar Negative Control siRNA (all from QIAGEN). Cells were grown in 24-well plates and Lipofectamine 2000 (Invitrogen) was used as a transfection agent. Knockdown after 72 hours was confirmed by Western blots and RT-PCR.

For α-actinin-4 overexpression, A549 cells (3×10^5^) were seeded in 35-mm glass-bottom dishes (MatTek, Ashland, MA) and transfected with an α-actinin-4-GFP or a GFP plasmid (Genecopoeia, Rockville, MD) using FuGene 6 Transfection Reagent (Roche Diagnostics).

### RT-PCR

RNA was prepared with the RNeasy Mini kit (QIAGEN) and treated with DNAse I (QIAGEN). cDNA was synthesized using the Superscript III first strand RT-PCR system (Invitrogen) with oligo-dT primers. Real-time PCR was performed on a Rotorgene 2000 instrument (Corbett Life Science, Sydney, Australia) using α-actinin-4 or α-actinin-1 QuantiTect Primer assays (QIAGEN) and a GAPDH assay (Applied Biosystems, Foster City, CA).

### F-Actin binding assay

An actin binding protein spin down assay kit (Cytoskeleton, Denver, CO) was used according to the manufacturer's instructions. Briefly, non-muscle actin was allowed to polymerize into F-actin. HAMLET was mixed with α-actinin from rabbit skeletal muscle in a 10∶1 molar ratio and incubated for 30 minutes. α-Actinin with or without HAMLET was mixed with actin and incubated for 30 minutes. F-Actin was isolated using centrifugation at 150 000×g for 1.5 hours at 24°C. The supernatant was removed and the pellet was dissolved in water. To the supernatants 6 µl 1M DTT and 12 µl LDS sample buffer (Invitrogen) was added and to the pellets 4 µl 1M DTT and 10 µl LDS sample buffer was added. The samples (20 µl of the supernatants and 15 µl of the pellets) were separated by SDS-PAGE on 4–12% Bis-Tris gels (Invitrogen) and stained by Blue silver [34].

### Statistical analysis

The Mann-Whitney test was performed with InStat software (version 3.06, GraphPad, San Diego, CA) in Fig. S4 and repeated measures ANOVA was used in Fig. 4B and 7D. The Student's T-test was used for Fig. 4D.

## Results

### HAMLET binds α-actinin-4 in cell extracts

**Figure 1:**
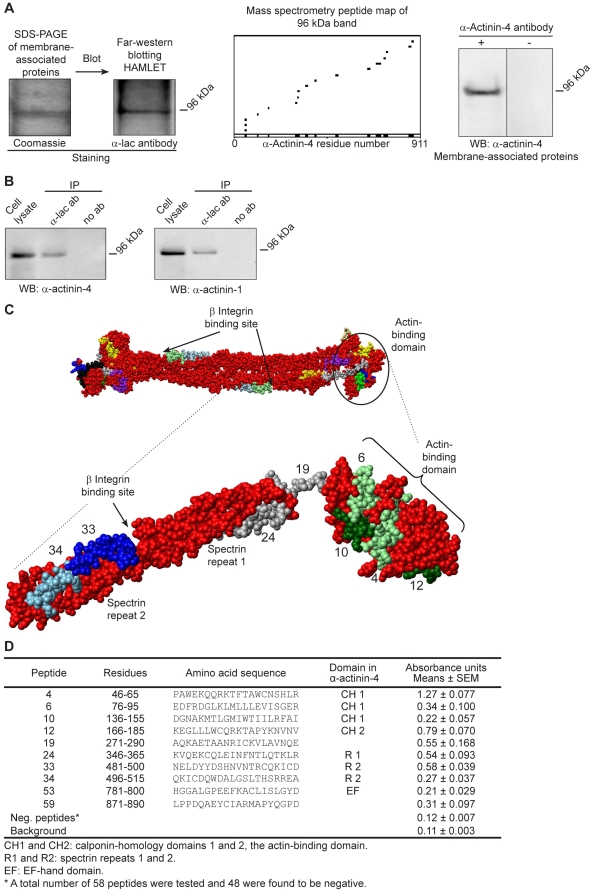
HAMLET binds to α-actinin-4. A) HAMLET was shown to bind to a 96 kDa protein in the membrane fraction of carcinoma cell extracts in a Far-Western blot with HAMLET. Bound HAMLET was detected using anti-α-lactalbumin antibodies. The 96 kDa band was identified as α-actinin-4 by mass spectrometry (Profound analysis). The identity of the band was confirmed in a Western blot of membrane-associated proteins stained with antibodies specific to α-actinin-4. B) Co-immunoprecipitation of HAMLET and α-actinin-4 and α-actinin-1 was detected in lysates from HAMLET-treated carcinoma cells (15 minutes, 35 µM). HAMLET and associated proteins were immunoprecipitated with anti-α-lactalbumin antibodies and lysates and precipitates were analyzed by Western blot. C) Peptide mapping of the interaction between HAMLET and α-actinin-4. The binding assay with HAMLET and synthetic, 20 amino acid long, α-actinin-4 peptides detected 10 positive peptides. Positive peptides in α-actinin-4 from the actin-binding domain are shown in green, one peptide in the hinge domain and one on the first spectrin repeat are shown in grey and two peptides near the β integrin-binding site in blue. The interacting peptides in the α-actinin antiparallel homodimer are shown on top (PDB code 1SJJ, visualized in MolMol). The enlargement illustrates HAMLET-binding peptides in an α-actinin monomer (residues 1-518). D) Table of α-actinin-4 peptides interacting with HAMLET and their corresponding residue numbers, amino acid sequences, domains in α-actinin-4 and absorbance units (means ± SEMs of 3 experiments).

To identify cellular targets involved in detachment, membrane-associated proteins were isolated from A549 carcinoma cells. After separation by SDS-PAGE and blotting, PVDF membranes were overlaid with HAMLET. Bound HAMLET was visualized using anti-α-lactalbumin and secondary HRP-conjugated antibodies (Fig. 1A, Fig. S1A). HAMLET was shown to bind to a 96 kDa band, which was identified by mass spectrometry as human α-actinin-4. Matching peptides covered 19% of the α-actinin-4 sequence (Fig. 1A, Table S1) and 10% of the α-actinin-1 sequence. The identity of the band was confirmed by Western blots using α-actinin-4-specific polyclonal antibodies (Fig. 1A). Further evidence of an interaction between α-actinin-4 and HAMLET in tumor cells was obtained by co-immunoprecipitation of extracts from HAMLET-treated A549 cells (15 minutes, 35 µM). Using α-lactalbumin antibodies for precipitation of HAMLET and α-actinin-4 antibodies for detection, a 96 kDa band was detected (Fig. 1B).

As α-actinin-1 and α-actinin-4 are structural homologues and share cellular functions, we subsequently examined if HAMLET also binds to α-actinin-1 in tumor cells. Membranes with the co-immunoprecipitation extracts were probed with an α-actinin-1 antibody, which detected a 96 kDa band at the same position as α-actinin-4 (Fig. 1B).

The results show that HAMLET interacts with α-actinins in cell extracts and in HAMLET-treated tumor cells.

### Peptide mapping of binding sites for HAMLET on α-actinin-4

The α-actinins are members of the spectrin superfamily, forming an anti-parallel homodimer with one N-terminal actin-binding domain at each end of the rod, a central rod domain with four spectrin-like repeats, and a C-terminal, calmodulin-like domain (Fig. 1C, [16], [35], [36]). The first and second spectrin repeats interact with the cytoplasmic domain of β integrins [7].

HAMLET binding sites were identified using a synthetic peptide library covering most of the α-actinin-4 sequence (each peptide was 20 amino acids long, with a 5 amino acid overlap). Binding of biotinylated peptides to HAMLET was quantified using streptavidin-alkaline phosphatase conjugates and interacting peptides were mapped onto a cryo-EM reconstruction representation of smooth muscle α-actinin (PDB code 1SJJ [26]) with MolMol software. Ten peptides with affinity for HAMLET were identified (Fig. 1C,D): peptides 4, 6, 10 and 12 in the actin-binding domain (CH1 and CH2), peptide 19 covering the neck region between the actin-binding and the central rod domain and peptide 24 corresponding to the first spectrin repeat on the central rod domain (R1). Peptides 4, 10, 19 and 24 all face the cavity between the actin-binding domain and the central rod domain, potentially forming a combined binding site for HAMLET. In addition, HAMLET bound to peptides 33 and 34 covering part of the proposed β integrin-binding site, which consists of three loops on the first and second spectrin repeat (R2) of α-actinin (Fig. 1C,D), [7]. Finally, HAMLET also bound to peptides 53 and 59 in the C-terminal part of α-actinin-4. According to the 3D structural model (Fig. 1C,D), all positive α-actinin-4 peptides are exposed in the monomer as well as in the anti-parallel homodimer. In the homodimer, peptides 53 and 59 of one monomer are localized close to HAMLET-binding peptides on the adjacent monomer and may form a combined binding site for HAMLET in the α-actinin-4 dimer (peptides 12, 19 and 24 for peptide 53 and peptides 4, 10 and 12 for peptide 59). The peptide map and visual inspection of the structures (PDB codes 1SJJ and 1HML [37]) suggested that HAMLET may potentially fit into the cavity between the actin-binding domain and the central rod domain, linked by the flexible neck domain. In addition, HAMLET may interact with part of the proposed β integrin-binding site between spectrin repeats 1 and 2 (Fig. 1C,D).

The results indicate that HAMLET recognizes distinct, functionally relevant α-actinin domains.

### HAMLET alters the distribution of α-actinin-4 in tumor cells

**Figure 2:**
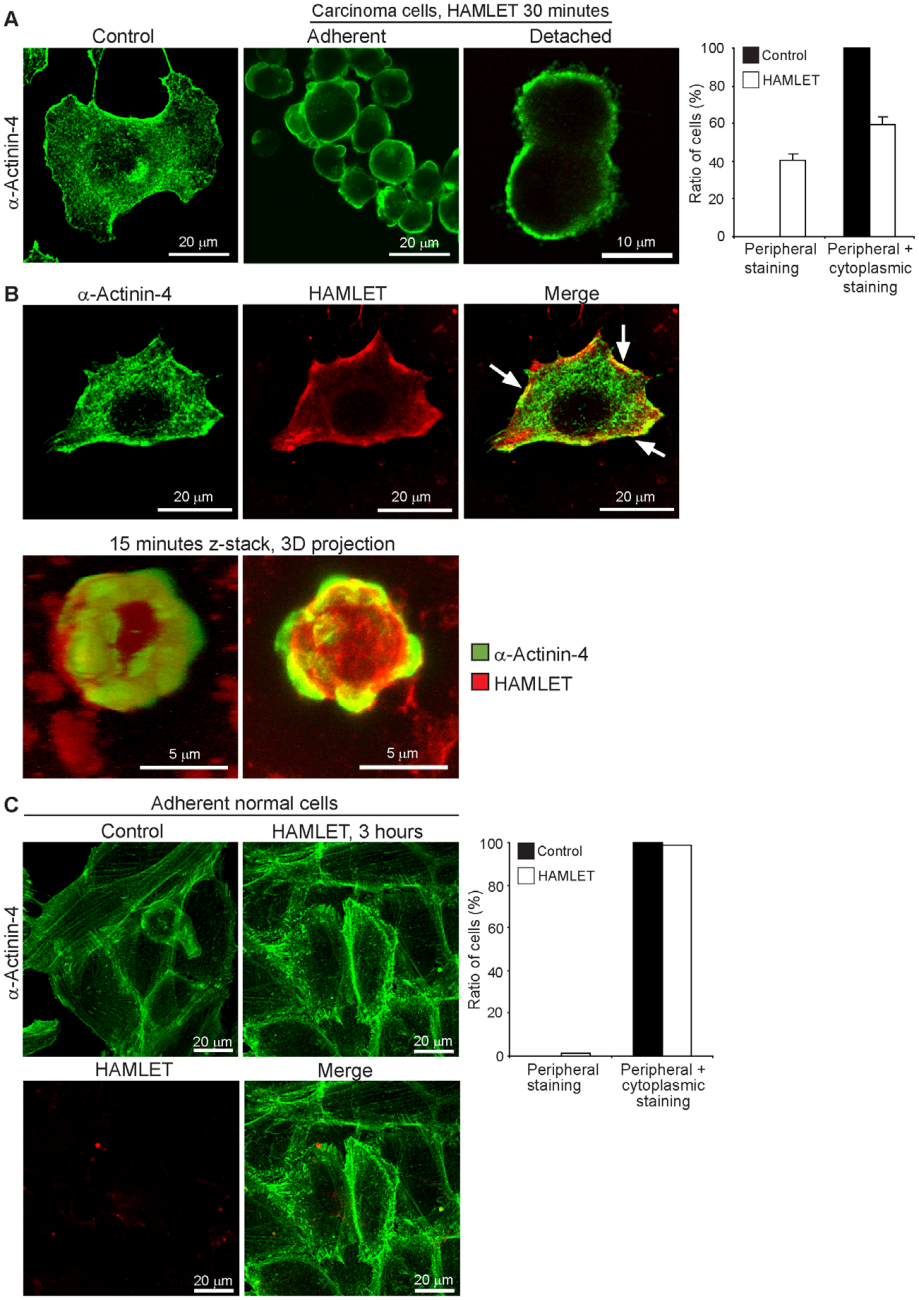
HAMLET alters the distribution of α-actinin-4 in tumor cells. Carcinoma cells and normal cells were cultured on glass slides, fixed and stained with antibodies specific for α-actinin-4 and examined by confocal microscopy. A) Granular α-actinin-4 staining along the cell membrane in untreated cells changed to uniform staining after HAMLET exposure of carcinoma cells and accumulation of α-actinin-4 in membrane blebs (35 µM, 30 minutes, detached cells 1 hour). Means + SEMs of 3 experiments (1 hour). B) Co-localization of HAMLET with α-actinin-4 in carcinoma cells. Carcinoma cells were treated with Alexa-Fluor 568-labeled HAMLET (red, 35 µM, 30 minutes) and stained with antibodies to human α-actinin-4 (green). HAMLET and α-actinin-4 co-localized at the cell periphery, especially where the cells started to contract (arrows). A Z-stack, 3D projection of a carcinoma cell (15 min) showed co-localization at the cell periphery where the cell is attached to the glass slide. C) Normal differentiated cells (HRTEC) showed accentuated membrane α-actinin-4 staining after HAMLET treatment (35 µM, 3 hours) but the actin stress fibres remained visible (means + SEMs of 2 experiments).

To further characterize the interaction between HAMLET and α-actinin-4 in tumor cells, adherent A549 cells were incubated with Alexa-Fluor 568-labeled HAMLET (35 µM, 1 hour), fixed, stained for α-actinin-4 and inspected by confocal microscopy. In untreated cells, α-actinin-4 staining in the cell periphery was patchy or granular, indicating that α-actinin-4 accumulated in the vicinity of certain membrane areas. After HAMLET treatment, α-actinin-4 membrane staining was more uniform, indicating a loss of membrane compartmentalization (Fig. 2A; for corresponding light images see Fig. S2A). Large amounts of HAMLET were internalized by the tumor cells and co-localization of HAMLET and α-actinin-4 was detected at the cell periphery already after 15 minutes (3D z-stack images in Fig. 2B, Movie S1, for corresponding light images see Fig. S2B). The cytoplasmic, trabecular staining pattern was lost after HAMLET treatment, suggesting a loss of cytoskeletal organization and a redistribution of α-actinin-4 to the cell periphery (Fig. 2A).

Normal differentiated cells (HRTEC) showed a patchy or granular α-actinin-4 staining pattern similar to the tumor cells and more pronounced cytoplasmic staining along stress fibers (Fig. 2C, for light images see Fig. S2C). Membrane staining was accentuated in response to HAMLET (35 µM, 3 hours), but the stress fibers remained visible even after HAMLET treatment and there was no change in cell morphology or evidence of detachment. The normal differentiated cells internalized very small amounts of HAMLET and membrane co-localization of HAMLET and α-actinin-4 was not observed (Fig. 2C).

The results show that HAMLET alters the cellular distribution of α-actinin-4 in carcinoma cells, but not in normal, differentiated cells and suggest that α-actinin-4 and HAMLET interact at the membrane level in tumor cells.

### HAMLET triggers rapid carcinoma cell detachment in vitro

**Figure 3:**
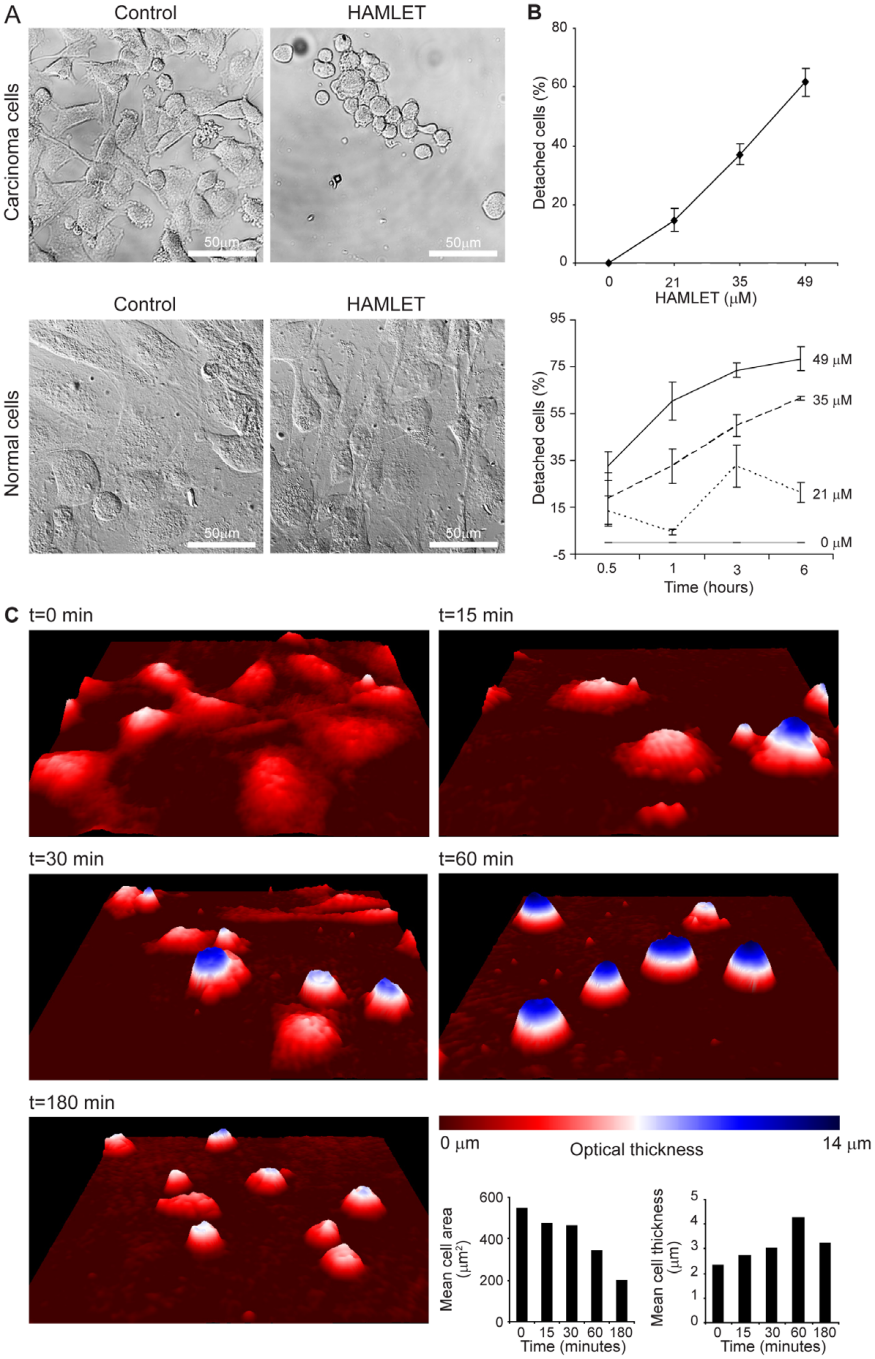
Quantification of cell detachment in response to HAMLET. A) A549 carcinoma cells or normal kidney cells in primary culture (HRTEC) were allowed to adhere to glass microscopy slides, treated with HAMLET (35 µM) and fixed. Cell confluence and morphology was examined by confocal microscopy. HAMLET caused rapid rounding up and detachment of carcinoma cells (30 minutes, upper panels) but normal cells did not change morphology or detach in response to HAMLET (1 hour, lower panels). B) The A549 cells detached in a time- and dose-dependent manner in response to HAMLET (means ± SEMs of 3 experiments). The top panel shows dose-dependent detachment after 3 hours of HAMLET treatment and the lower panel shows both dose- and time-dependent detachment. Detachment was quantified by measuring hexosaminidase activity. C) The morphological changes in tumor cells after HAMLET treatment were examined by real time holographic imaging (35 µM; HoloStudio™). After 15 minutes, membrane blebbing was observed followed by rounding up after 30 minutes and 1 hour. After 3 hours, the cells had shrunk and the flattened diffuse signal indicates loss of viability. The mean cell area and optical thickness was measured at the different time points using the HoloStudio™.

To address how HAMLET influences cell morphology and detachment, several techniques were used. Adherent human lung carcinoma or normal, differentiated cells were exposed to HAMLET (35 µM) and by light microscopy, a reduction in confluence of adherent cell layers of carcinoma cells was observed after 30 minutes, suggesting detachment (Fig. 3A). In addition, the adherent cells changed morphology, from a distended flattened appearance with pseudopods to a fully rounded shape with occasional surface blebs, suggesting a loss of cytoskeletal integrity. Detached cells, which were harvested from the supernatant, retained this rounded morphology (data not shown). Normal differentiated cells, in contrast, remained adherent with distended morphology after 1 hour in the presence of HAMLET (35 µM, Fig. 3A).

Cell detachment was further quantified as the loss of hexosaminidase activity. Hexosaminidase is a lysosomal enzyme catalyzing the degradation of glycosylated cellular constituents and due to its widespread occurrence in cells of different origins, hexosaminidase has been extensively used to measure cell numbers [28]. Exposure of adherent carcinoma cells to HAMLET triggered a dose- and time-dependent increase in cell detachment (Fig. 3B). After 3 hours exposure to 35 µM of HAMLET, 37% of the cells had detached, compared to 62% at 49 µM. Cell detachment increased over time, from 33% after 1 hour to 62% after 6 hours exposure to 35 µM of HAMLET. The cell viability of the detached cells was also examined. After 3 hours of HAMLET treatment, cells treated with 7 and 21 µM of HAMLET that had detached showed 70 and 59% viability, suggesting that detachment preceded death. At higher HAMLET concentrations (35 µM) detached and adherent cells died rapidly (5% viability of detached cells, Fig. S3).

Holographic imaging records 3D information of an unfixed object in real time, based on interfering wave fronts from a laser and was used to further characterize the cellular changes that accompany the detachment process (Fig. 3C). After 15 minutes exposure to HAMLET (35 µM), adherent human lung carcinoma cells showed a reduction in surface area and an increase in thickness and with evidence of membrane blebbing. After 1 hour, the cells became smaller (from 547 to 341 µm^2^) and thicker (from 2.33 to 4.27 µm) with a more rounded outline. The mean eccentricity was reduced from 0.785 to 0.666 after HAMLET exposure (1 hour, 35 µM).

The results show that HAMLET triggers rapid detachment of tumor cells, but not of normal differentiated cells. In addition, remaining tumor cells changed from a distended and flattened to a rounded shape, consistent with loss of cytoskeletal organization.

### Inhibition of cell detachment by α-actinin-4 peptides

The synthetic α-actinin-4 peptides used to map the interaction of HAMLET with α-actinin-4 were also tested for inhibition of cell detachment. HAMLET was pre-incubated with each peptide for one hour, the mixture was added to A549 lung carcinoma cells and cell morphology and detachment were examined (Fig. S4). Peptides 12 and 34 reduced the rounding up (12: median values 32% vs. 70%, ns; 34: median values 38% vs. 70%, ** p<0.01) and the detachment of the carcinoma cells (12: mean values 18% vs. 11%; 34: mean values 22% vs. 11%, ns). Peptides 4 and 24 also inhibited cells from rounding up (4: median values 47% vs. 70%, ns; 24: median values 27% vs. 70%, ** p<0.01) but the effects might be non-specific due to aggregate formation of HAMLET and peptide.

The results are consistent with the specificity of HAMLET for α-actinin domains involved in cell adhesion.

### Effects of α-actinin expression on cell detachment in response to HAMLET

**Figure 4:**
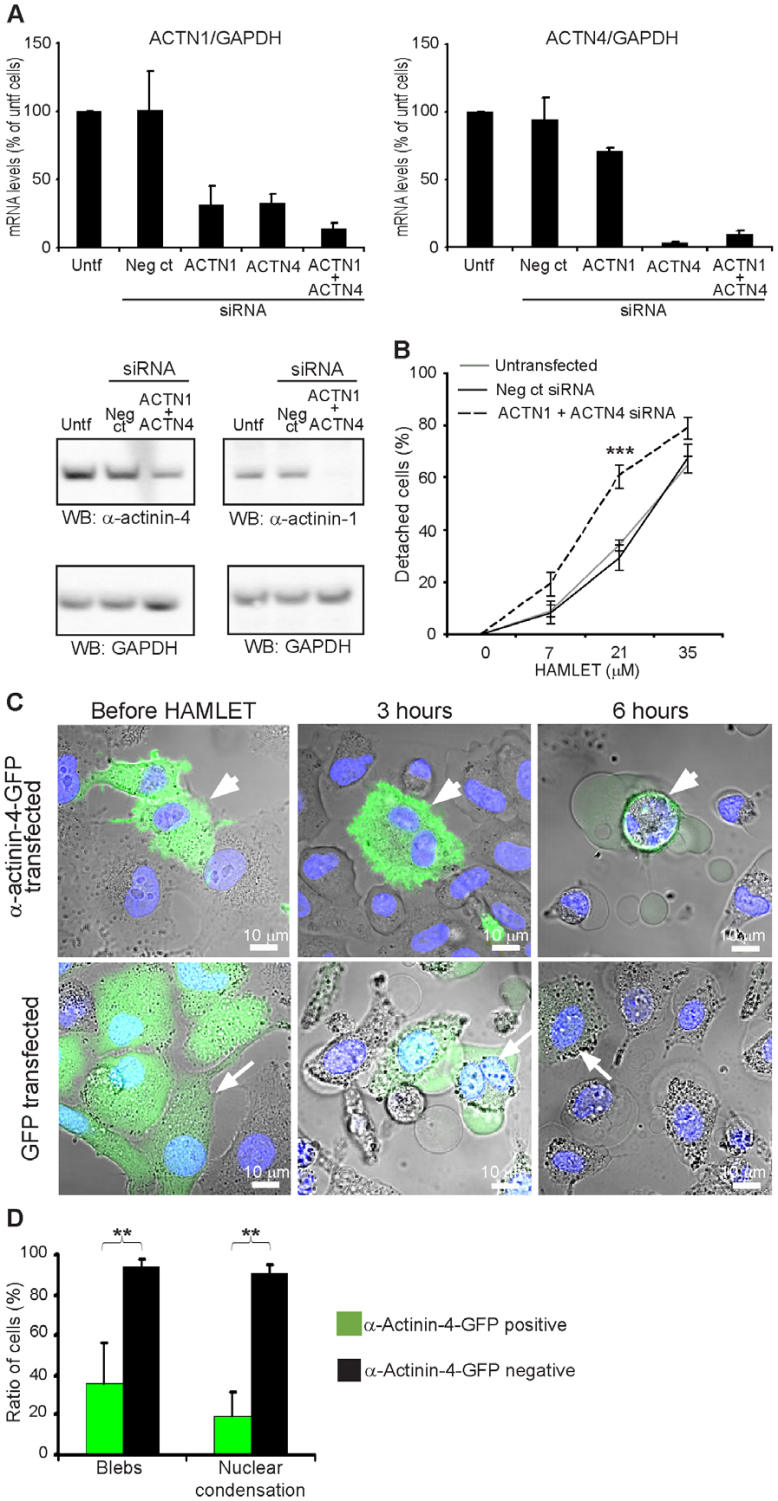
Altered α-actinin expression modifies cell detachment. A) α-Actinin siRNA knockdown was quantified by RT-PCR (means + SEM of 4 experiments) and Western blot. B) The hexosaminidase activity assay was used to examine the cell detachment after siRNA-treatment. In response to HAMLET, tumor cell detachment was increased in cells with low α-actinin levels compared to cells transfected with negative control-siRNA or untransfected cells (means ± SEMs of 4 experiments, *** p<0.001). C) HAMLET response of cells overexpressing α-actinin-4-GFP was studied by live cell confocal microscopy. Rounding up in response to HAMLET (21 µM) was delayed compared to cells transfected with the GFP plasmid and cells without the α-actinin-4-GFP plasmid. D) Overexpression of α-actinin-4-GFP in A549 cells delays blebbing and nuclear condensation caused by HAMLET (21 µM, 3 hours, means + SDs of 3 experiments, ** p<0.01).

To examine if inhibition of α-actinin expression modifies tumor cell detachment in response to HAMLET, A549 carcinoma cells were transfected with α-actinin-4- and α-actinin-1-specific siRNAs and knockdown was confirmed by RT-PCR and Western blots (Fig. 4A) and detachment was quantified using the hexosaminidase assay. The α-actinin-4- and α-actinin-1-specific siRNA-transfected cell population detached to a greater extent in response to HAMLET than non-transfected or control siRNA-transfected cells (61% vs. 33%, *** p<0.001, 21 µM, 3 hours, Fig. 4B). Cells transfected with both α-actinin-4- and α-actinin-1 siRNAs showed a more pronounced detachment response to HAMLET (Fig. S5) than cells treated with either siRNA alone. Furthermore, by holographic imaging, α-actinin siRNA-treated carcinoma cells were shown to be more round, to cover a smaller surface area and have a greater thickness than control siRNA-transfected cells. In response to HAMLET, cells with low α-actinin levels rounded up faster and more (lower eccentricity) than control siRNA-transfected cells (data not shown).

To examine if α-actinin-4 overexpression reduces detachment in response to HAMLET in carcinoma cells, A549 cells were subsequently transfected with an α-actinin-4-GFP plasmid and α-actinin-4-GFP expression was detected by live cell confocal imaging. Cells transfected with an insert-free GFP control plasmid were used as controls. Seventy-two hours after transfection, α-actinin-4-GFP expression levels varied in the transfected cell population, as determined by the GFP staining intensity (Fig. 4C). There was a marked difference in the response to HAMLET (21 µM, 3 hours) between α-actinin-4-GFP overexpressing cells and cells with normal α-actinin-4 levels in the same population. Cells overexpressing α-actinin-4-GFP were larger than surrounding cells, with a flattened morphology and their morphology remained unchanged after 3 hours of HAMLET-treatment (Fig. 4C). In the same sample, cells without detectable α-actinin-4-GFP expression rounded up in response to HAMLET and blebbing and nuclear condensation was observed. Similar changes were observed in the GFP-control plasmid-transfected and untransfected control cells (Fig. 4C). Cells which overexpressed α-actinin-4-GFP showed reduced blebbing and nuclear condensation (21 µM, 3 hours, ** p<0.01, Fig. 4D) compared to cells with normal α-actinin-4 levels. These are two characteristics of the cell death response to HAMLET [18]. After 6 hours exposure to HAMLET, morphological changes occurred also in α-actinin-4-GFP overexpressing cells, however.

The results show that α-actinin expression levels modify the change in carcinoma cell morphology as well as detachment in response to HAMLET.

### HAMLET changes β1 integrin distribution and perturbs focal adhesion kinase signaling

**Figure 5:**
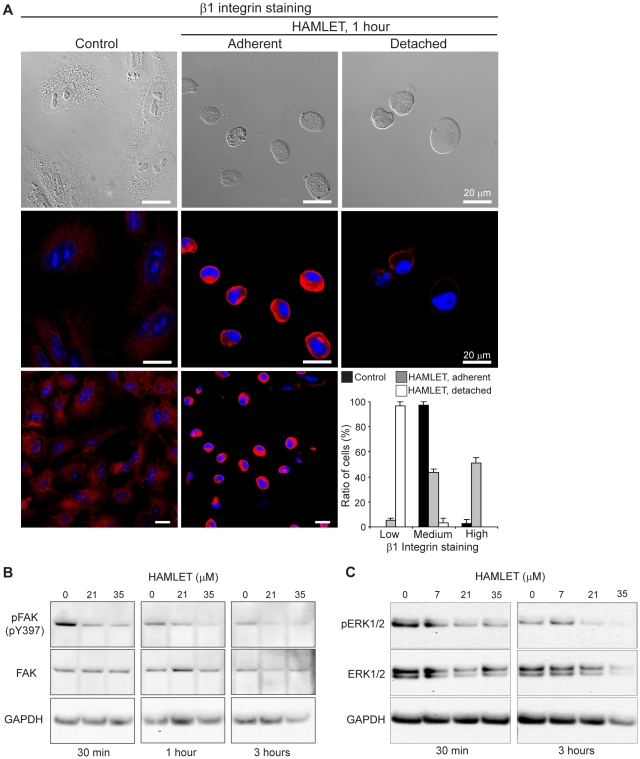
HAMLET changes β1 integrin distribution and disrupts FAK/ERK signaling. Carcinoma cells were cultured on glass slides, treated with HAMLET and stained with antibodies specific for β1 integrins and examined by confocal microscopy. A) Reduction of β1 integrin staining following HAMLET treatment (35 µM, 1 hour). Untreated carcinoma cells showed a diffuse staining pattern. After HAMLET treatment (35 µM, 1 hour) the adherent cells remained β1 integrin-positive while the detached cells showed reduced β1 integrin staining. Adherent untreated and HAMLET-treated cells and detached cells are shown with the corresponding light microscopy image. Means + SEMs of 3 experiments. B) HAMLET reduces FAK phosphorylation (30 minutes) and induces FAK cleavage (3 hours) as shown by western blots using FAK and phospho-FAK (pTyr397) antibodies. GAPDH was used as loading control. C) HAMLET reduces ERK1/2 phosphorylation (30 minutes) and induces ERK1/2 degradation (3 hours) as shown by western blots using ERK1/2 and phospho-ERK1/2 antibodies. GAPDH was used as loading control.

The effect of HAMLET on β1 integrin localization was examined by confocal microscopy in A549 carcinoma cells (Fig. 5A). Untreated, adherent carcinoma cells showed diffuse, mainly cytoplasmic β1 integrin staining. After HAMLET exposure (35 µM, 1 hour), remaining adherent cells had rounded up and showed an increase in cytoplasmic β1 integrin staining, with accumulation in large cytoplasmic aggregates in certain cells (Fig. 5A). The detached, rounded cells showed weak β1-integrin staining in the cell periphery.

Loss of β1 integrin binding to the extracellular matrix has previously been shown to perturb FAK signaling and the MAPK/ERK kinase pathway downstream of FAK is important for cell survival and proliferation [38], [39]. The effect of HAMLET-induced detachment on FAK and ERK1/2 phosphorylation was examined by Western blots, using antibodies specific for phosphorylated FAK (pTyr397) and phosphorylated ERK1/2 (pThr202/Tyr204, ERK1 and pThr185/Tyr187, ERK2). Adherent A549 carcinoma cells treated with HAMLET showed a reduction in FAK phosphorylation after 30 minutes (21 and 35 µM) that was sustained for 3 hours. Cleavage of FAK was also detected after 3 hours (Fig. 5B). HAMLET-induced detachment also caused a reduction in ERK1/2 phosphorylation in A549 carcinoma cells after 30 minutes and 3 hours (21 and 35 µM) and reduced total ERK1/2 levels after 3 hours (Fig. 5C). Cells detached by EDTA treatment showed a slight reduction in FAK phosphorylation but a slight increase in ERK phosphorylation (Fig. S6).

The results suggest that detachment caused by HAMLET involves the loss of integrin function and perturbs FAK and ERK1/2 signaling.

### Interactions with actin and β1 integrins

**Figure 6:**
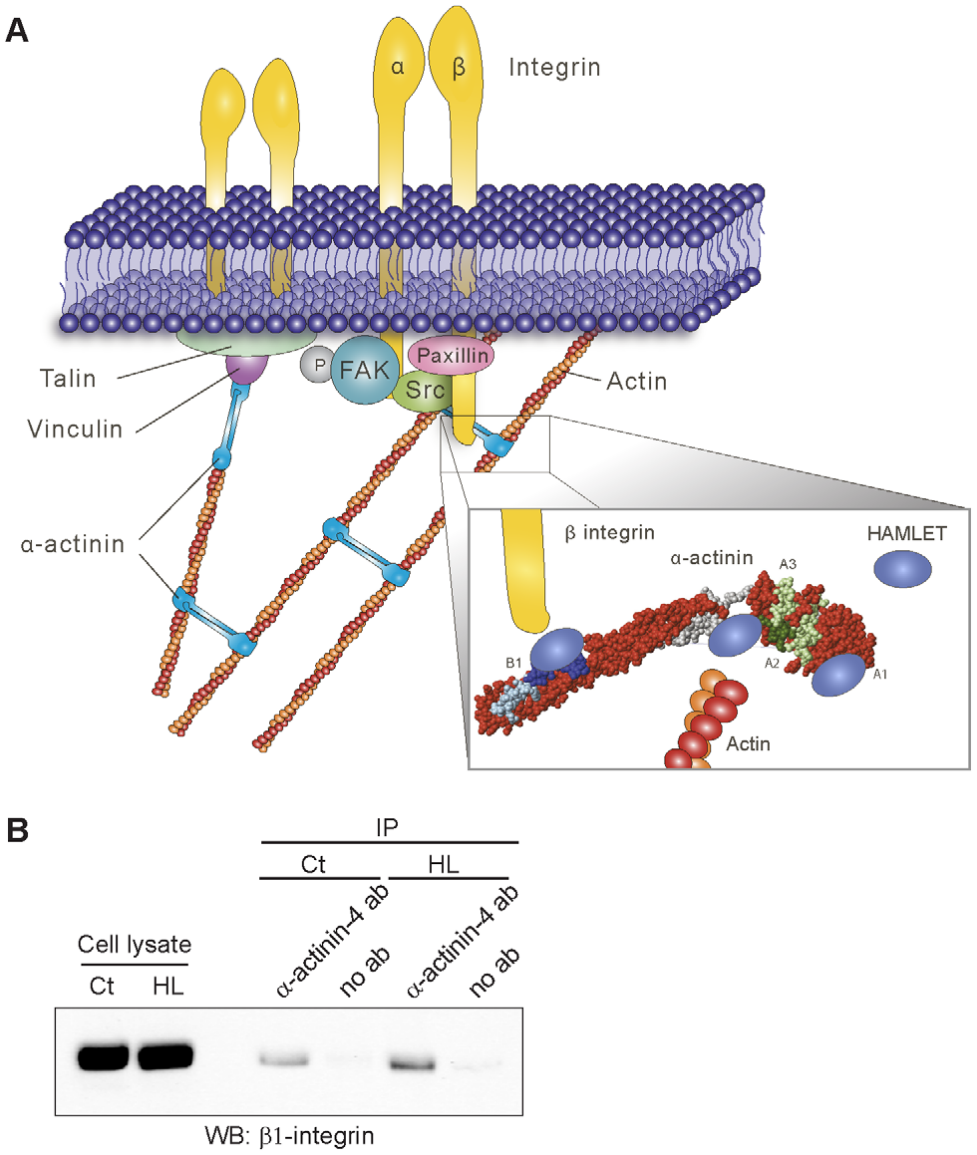
Schematic model of HAMLET-α-actinin interactions. A) The figure is based on information available in MolMol (PDB codes 1SJJ and 1HML) and on results from the peptide-binding assay and not on actual three-dimensional structural data of HAMLET bound to α-actinin-4. α-Actinin forms a bridge between the cytoskeleton and the integrins through the actin- and β integrin-binding sites but also through vinculin and talin. By binding to α-actinin, HAMLET may disrupt focal adhesions (enlargement, lower right). HAMLET interacts with the actin-binding domain of α-actinin and fits into the pocket formed between the actin-binding domain and the first spectrin repeat. In addition, HAMLET binds adjacent to the β integrin-binding site. B) Cell extracts from untreated or HAMLET-treated cells were used for co-immunoprecipitation with anti-α-actinin-4 antibodies. Blots stained with anti-β1 integrin antibodies show that α-actinin-4 and β1 integrin still interact with each other in the presence of HAMLET.

The changes in carcinoma cell morphology and behavior suggested that cytoskeletal rearrangements might be involved, consistent with the function of α-actinin-1 and -4 as cytoskeletal crosslinkers and the binding of HAMLET to epitopes within the previously identified actin- and β1 integrin-binding sites in the α-actinin-1 and -4 isoforms (Fig. 1 and Fig. 6). In an attempt to directly address if HAMLET disrupts the interaction between α-actinin and F-actin, an actin binding protein spin down assay was used. F-actin was isolated by ultra-centrifugation and proteins bound to the filaments were detected by SDS-PAGE. α-Actinin was shown to bind to F-actin both in the absence and presence of HAMLET (Fig. S7), indicating that HAMLET did not disrupt the interaction between α-actinin and F-actin under these conditions. In addition, in an ELISA-based method, HAMLET was shown to bind to α-actinin but binding of F-actin to α-actinin could not be detected and for this reason inhibition by HAMLET could not be measured. The conditions in the spin down assay are likely to be unfavorable for maintaining the properties of the HAMLET complex. For example, HAMLET triggers a rapid increase in intracellular Ca^2+^ [18] and high Ca^2+^ levels reduce the binding between α-actinin and F-actin [16]. Such a modification step might be required before HAMLET can compete with F-actin for binding to α-actinin. The lack of F-actin binding in the ELISA may be due to missing cofactors, wrong pH or other unfavorable *in vitro* conditions such that the actin filaments are disrupted under conditions where HAMLET binds to α-actinin.

To examine if HAMLET perturbs the interaction between α-actinin-4 and β1 integrins, total cell extracts from adherent carcinoma cells were precipitated using anti-α-actinin-4 antibodies, before or after HAMLET treatment (35 µM, 3 hours). Blots stained with anti-β1 integrin antibodies show that α-actinin-4 and β1 integrin still interact with each other in the presence of HAMLET (Fig. 6B). This may indicate that binding of HAMLET to peptide 33, which only partly covers the β1 integrin binding site on α-actinin, does not directly hinder β1 integrin binding.

### Detachment and cell viability

**Figure 7:**
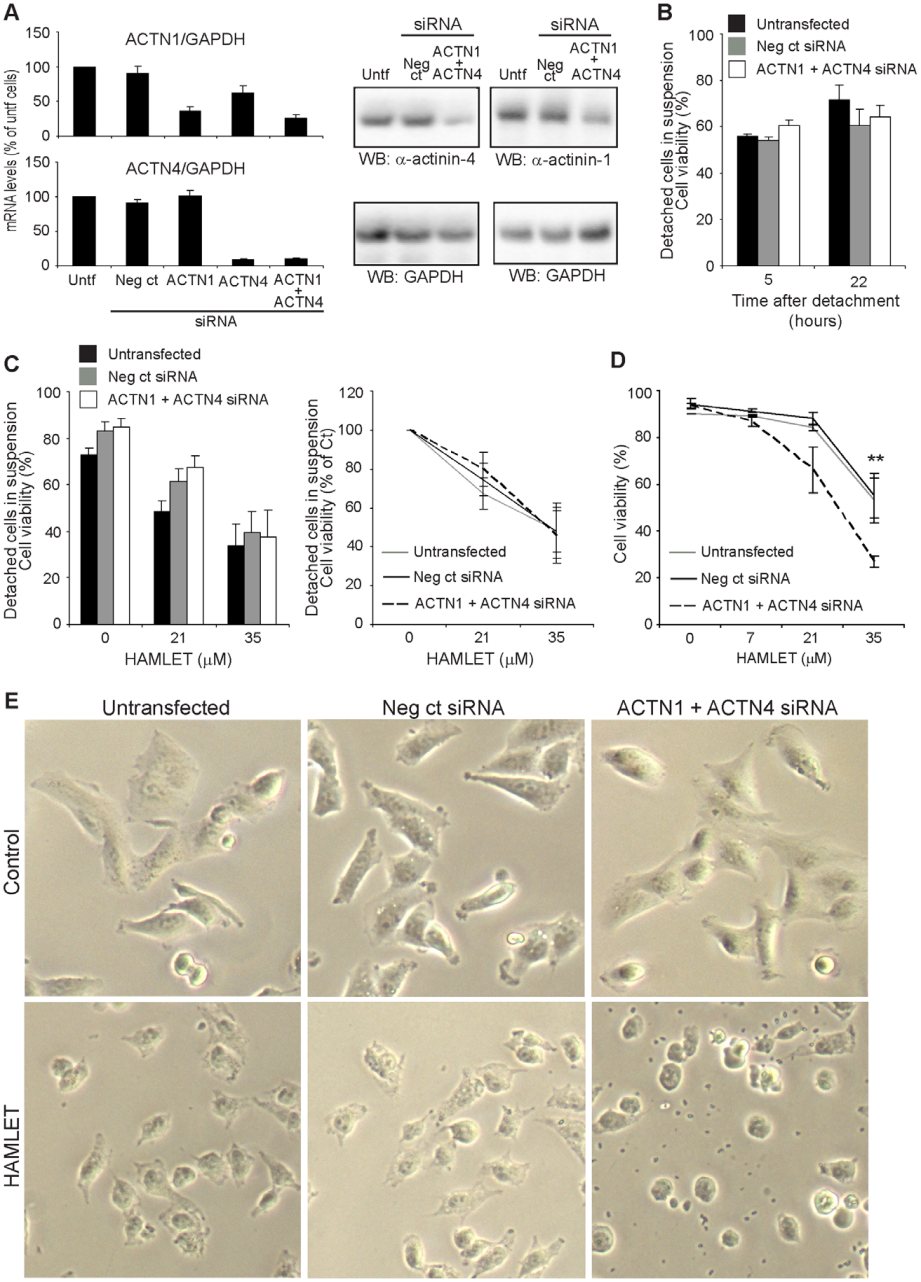
Cell detachment and death. A) α-Actinin siRNA knockdown was quantified by RT-PCR (means + SEMs of 4 experiments) and Western blot. B) Cells that were kept in suspension for 22 hours showed the same viability indepently of α-actinin levels by trypan blue exclusion. C) HAMLET treatment of cells in suspension (non-transfected, control siRNA-transfected or α-actinin siRNA-transfected) did not show a difference in sensitivity as determined by trypan blue exclusion. The left diagram shows the absolute numbers while the right diagram shows cell death in % of control. D) By trypan blue exclusion, tumor cell death was increased in adherent cells with low α-actinin levels (means ± SEMs of 4 experiments, ** p<0.01). E) By light microscopy, the cells with low α-actinin levels show a more rapid change in morphology in response to HAMLET than the cells with normal α-actinin levels. The rounding up was more pronounced in cells with low α-actinin levels.

To investigate the relationship between α-actinin expression, detachment and death, carcinoma cells were treated with α-actinin-1 and -4 specific siRNAs for 72 hours and knockdown of α-actinin-1 and -4 was confirmed by RT-PCR and Western blots (Fig. 7A). The cells were detached, kept in suspension and cell viability was quantified as trypan blue exclusion. After 22 hours in suspension, no difference in cell viability was detected between the cells with low α-actinin expression and non-transfected or control siRNA-transfected cells (Fig. 7B). In parallel, the sensitivity of the detached cells to HAMLET (35 µM, 3 hours) was quantified as a function of α-actinin expression. The detached cells rapidly lost viability (Fig. 7C) but no significant difference in cell death was detected between α-actinin-1 and -4 siRNA-transfected cells with low α-actinin levels and transfected control cells with normal α-actinin levels (Fig. 7C).

The results suggest that the rapid cell death caused by HAMLET does not depend on α-actinin expression. Furthermore, detachment alone did not cause significant cell death during the experiment.

To further examine if cell death and detachment may be distinguished in adherent cells, the loss of viability was compared between adherent α-actinin-1 and -4 specific siRNA-transfected and control cells. Cells with low α-actinin levels were shown to die to a greater extent than non-transfected or control siRNA-transfected cells (53% vs. 27%, ** p<0.01, 35 µM, 3 hours, Fig. 7D). Cells transfected with both α-actinin-4- and α-actinin-1-specific siRNAs showed a more pronounced cell death response to HAMLET (Fig. S5) than cells treated with either siRNA alone and cells with low α-actinin levels also rounded up to a greater extent than the cells with normal α-actinin levels (Fig. 7E). The results indicate that α-actinin expression modifies the death response to HAMLET of adherent cells.

## Discussion

HAMLET triggers rapid carcinoma cell detachment *in vitro* and in bladder cancer patients, after topical administration [20]. This study examined the molecular basis for carcinoma cell detachment and identified α-actinins as HAMLET targets. α-Actinins link integrins to the actin cytoskeleton by acting as a scaffold between actin and β integrins, maintaining cytoskeletal architecture and cell adhesion [3], [9], [14]. Using synthetic α-actinin-4 peptides, HAMLET binding sites were mapped to the N-terminal actin-binding domain and the proposed β integrin-binding central rod domain between spectrin repeats 1 and 2, suggesting that HAMLET might impair these essential interactions, [5], [6], [10]. Consistent with this effect, HAMLET caused a rapid change from extended to round morphology, suggesting a disruption of cytoskeletal architecture. In addition, the cells lost β1 integrin staining and inhibited FAK and MEK/ERK signaling. The interaction of HAMLET with different α-actinin domains might thus act to destabilize tumor cell adhesion.

In the present study, normal cells in primary culture were shown not to detach or change their morphology in response to HAMLET under conditions where cancer cells underwent such changes. α-Actinin-4 is present both in tumor cells and normal differentiated cells, suggesting that α-actinin-4 *per se* is not a cellular target determining the increased HAMLET sensitivity of carcinoma cells. The role of α-actinin-4/α-actinin-1 in cancer development and progression remains ambiguous and it is unclear if α-actinin-4/α-actinin-1 might serve as cancer cell targets. α-Actinin-4 overexpression correlates with increased tumor invasiveness and metastasis in pancreatic, esophageal, ovarian and lung carcinomas [40], [41], [42]. In contrast, its expression was reduced in neuroblastomas and prostate cancers and a mutated form of α-actinin-4 with decreased actin binding affinity promoted lung cancer [43], [44], [45]. Human autosomal dominant, gain of function mutations and a loss of function mouse knockout both caused focal and segmental glomerulosclerosis but no systemic tumorigenicity has been observed [46], [47], suggesting that α-actinin-4 variation *per se* may not be oncogenic. Recently it was suggested that α-actinin-1 was involved in tumorigenesis as its inhibition by RNAi in colon carcinioma cells enhanced tumor-free survival [48]. In addition Quick and Skalli proposed that the levels of α-actinin-1 and -4 are modulated in astrocytomas [15].

Several actin-binding sites have been mapped in the different α-actinin isoforms. Honda et al. proposed amino acids 111-125 as an actin-binding site in α-actinin-4 [9], corresponding to peptide 10 in our synthetic α-actinin-4 peptide library. Franzot et al. proposed that residues 48–57, 123–147 and 153–172 form three independent actin-binding sites in α-actinin-3 [6], corresponding to synthetic α-actinin-4 peptides 4, 10 and 12, which all bound to HAMLET. Based on these comparisons, we propose that HAMLET binds to epitopes within the previously identified actin-binding sites in the α-actinin-1 and -4 isoforms. The amino acids covered by the positive peptides are also present in α-actinin-1 but with other residue numbers. The amino acid sequences covered by peptides 4, 6, 19 and 24 are identical in α-actinin-4 and -1 and the sequences covered by peptides 10 and 12 differ by one residue, suggesting that the interactions of HAMLET with the actin-binding sites may be similar in both isoforms. While molecular details of these interactions remain to be defined, the peptide map and visual inspection of the structures (PDB codes 1SJJ and 1HML) suggested that HAMLET may potentially fit into the cavity between the actin-binding domain and the central rod domain, linked by the flexible neck domain (Fig. 6A). Peptide 33 differed by three and peptide 34 by four residues, however, suggesting that this region may have undergone divergent evolution and that HAMLET interactions with the spectrin repeats in α-actinin should be verified separately.

The involvement of α-actinin-4 and -1 in cell detachment was supported by siRNA transfection as inhibition of α-actinin-4 and -1 expression enhanced cell detachment and overexpression of α-actinin-4 protected carcinoma cells from detaching in response to HAMLET. In addition, overexpression of α-actinin-4-GFP delayed the blebbing and nuclear condensation, which are characteristics of HAMLET-induced cell death [18], seen in the cells with normal α-actinin-4 levels. Detachment was identified as a parameter independent of death, however, as transfected cells in suspension, expressing low α-actinin-4 and -1 levels did not die to a greater extent than untransfected cells. These cells responded to HAMLET with similar rapid kinetics as cells with normal α-actinin-expression, suggesting that α-actinin-4 and -1 are not involved in the cell death response *per se*. In adherent cells with low α-actinin levels, no increase in spontaneous cell death was observed but cells with low α-actinin levels were more sensitive to HAMLET-induced death than the cells with normal α-actinin levels, consistent with the broader effects of α-actinins on receptors and signaling.

Cell detachment has been suggested to promote cell death, as cells in suspension are at a disadvantage as targets for growth factors as well as essential nutritional components. Detachment from the extracellular matrix may induce anoikis with apoptosis-like cell characteristics in normal cells, whereas most tumor cells proliferate also in the absence of anchorage-dependent signaling [1], [49]. On the other hand, disruption of the cytoskeletal architecture in carcinoma cells induces amorphism; a form of apoptosis. Both mechanisms are controlled by the mitochondria through activation of the Bcl-2 protein family and death is executed by effector caspases [1], [2]. HAMLET has previously been shown to trigger apoptosis and to interact directly with mitochondria, leading to cytochrome c release, followed by caspase activation, DNA fragmentation and annexin staining [50], [51]. Cell death in response to HAMLET is independent of this pathway, however, as caspase 3-negative or Bcl-2 and Bcl-xl over-expressing cells die with rapid kinetics and caspase inhibition does not alter survival ([50], unpublished data). The mechanisms of HAMLET-induced detachment and death observed in this study are thus unrelated to apotosis, anoikis or amorphism.

Cell detachment has also been studied as an essential early step in the metastatic spread of carcinoma cells. The hallmarks of metastatic cells, include their ability to break through the basal lamina, infiltrate blood vessels, exit the blood vessels, and form new tumors, through finely regulated biomechanical interactions [52], [53]. Recently, large non-apoptotic blebs, with an amoeboid, highly motile cellular phenotype were described and molecular studies suggested that Akt signaling is involved and possibly small Rho-GTPases [54]. The morphological changes of HAMLET-treated cells included the formation of large blebs, suggesting that similar molecular mechanisms might be involved. Our *in vivo* studies have shown that HAMLET triggers shedding of human cancer cells into the lumen in the case of urinary bladder cancers and that the majority of the shed cells are dead [20]. Thus, HAMLET is not likely to increase the risk for metastases, as tumor cells die and detach with similar, rapid kinetics.

Maintaining the balance between the proliferative MEK/ERK1/2 pathway and the apoptosis-promoting p38 and JNK pathways is essential to control cell survival [55]. In a genome-wide transcriptomic analysis of HAMLET-treated cells, we recently identified the p38 pathway as a top-scoring pathway in HAMLET-treated cells and showed that p38 inhibition delays cell death (Storm et al, manuscript). This mode of HAMLET action is consistent with the inhibition of MEK/ERK signaling observed in the present study. Preliminary observations suggest that inhibition of p38 also reduces tumor cell detachment in response to HAMLET. Furthermore, the Rho family of small GTPases influence the association of α-actinins with lamellipodia as well as cell death and detachment in response to HAMLET (unpublished data) and α-actinin-4 silencing decreases RhoA mRNA levels [15], [56]. In addition, α-actinins associate with ion channels such as the L-type calcium channel and the Kv1.4 and Kv1.5 potassium channels influencing the activation of ion channels and intracellular Ca^2+^ levels, which modulate the actin-binding properties of α-actinins [16]. HAMLET activates rapid ion fluxes and increases intracellular Ca^2+^ levels in cancer cells [18] and ion channel activation has recently been identified as a key element of the lethal response to HAMLET (Storm et al, manuscript). The rapid and strong detachment of tumor cells exposed to HAMLET might thus reflect the combined activation of ion channels, small GTPases and p38 signaling and α-actinin may act by modulating several of these effects.

## Supporting Information

Figure S1 **HAMLET binds to α-actinin-4.** HAMLET was shown to bind to a 96 kDa protein in the membrane fraction of carcinoma cell extracts in a Far-Western blot with HAMLET. Bound HAMLET was detected using anti-α-lactalbumin antibodies. The 96 kDa band was identified as α-actinin-4 by mass spectrometry (Profound analysis).
(TIF)

Figure S2 **Light images.** Light images corresponding to Figure 2. Tumor cells underwent morphological changes after HAMLET treatment while normal differentiated cells remained adherent with a flattened and distended morphology.
(TIF)

Figure S3 **Cell viability in response to HAMLET.** A) Adherent cells were treated with HAMLET and the cell viability of detached cells was quantified by trypan blue exclusion. After 3 hours of HAMLET treatment, cells treated with 7 and 21 µM of HAMLET that had detached showed 70 and 59% viability while the cells treated with 35 µM of HAMLET showed 5% viability (means + SEMs of 3 experiments). B) The cell viability of the remaining adherent cells was also quantified by trypan blue exclusion (means + SEMs of 2 experiments).
(TIF)

Figure S4 **Inhibition of cell detachment by α-actinin-4 peptides.** A, B) HAMLET was pre-incubated with the ten α-actinin-4 peptides that bound to HAMLET in the peptide-binding assay. The pre-incubated mixture or HAMLET alone was added to carcinoma cells grown on glass slides and the morphology (A) and detachment (B) were analyzed by light microscopy. Peptides 12 and 34 reduced the rounding up and the detachment of the carcinoma cells. Peptides 4 and 24 also inhibited cells from rounding up but the effects might be non-specific due to aggregate formation of HAMLET and peptide (A: 4 experiments; B: means + SEMs of 4 experiments; ** p<0.01).
(TIF)

Figure S5 **Altered α-actinin expression modifies cell detachment.** A, B) α-Actinin siRNA knockdown was quantified by RT-PCR (A, means + SEMs of 3 experiments) and Western blot (B). C) The hexosaminidase activity assay was used to examine cell detachment after siRNA-treatment. In response to HAMLET tumor cell detachment was increased in cells with low α-actinin levels compared to cells transfected with negative control-siRNA or untransfected cells (means ± SEMs of 3 experiments). Cells transfected with both α-actinin-4- and α-actinin-1-specific siRNA showed a more pronounced detachment in response to HAMLET than cells treated with either siRNA alone. D) By Trypan blue exclusion, tumor cell death was increased in adherent cells with low α-actinin levels (means ± SEMs of 3 experiments). Cells transfected with both α-actinin-4- and α-actinin-1-specific siRNA showed a more pronounced cell death in response to HAMLET than cells treated with either siRNA alone.
(TIF)

Figure S6 **Detachment and FAK/ERK phosphorylation.** A) Cells detached by EDTA treatment showed a slight reduction in FAK phosphorylation as shown by western blots using FAK and phospho-FAK (pTyr397) antibodies. GAPDH was used as loading control. B) Cells detached by EDTA treatment showed a slight increase in ERK phosphorylation as shown by western blots using ERK1/2 and phospho-ERK1/2 antibodies. GAPDH was used as loading control.
(TIF)

Figure S7 **Actin-binding spin down assay.** An actin-binding spin down assay was performed to examine if HAMLET disrupts the interaction of α-actinin with F-actin. After ultracentrifugation, F-actin forms a pellet (P) and actin-binding proteins are also detected in these fractions. Proteins that do not interact with actin remain in the supernatant (S).
(TIF)

Table S1 Identification of α-actinin-4 by mass spectrometry.
(DOC)

Movie S1 **Co-localization of α-actinin-4 (green) and Alexa 568-labelled HAMLET (red) in the periphery of tumor cells.** 3D projection of images collected as a z-stack. Pictures were taken with a 0.37 μm interval and a 3D projection was made with the LSM510 software. Frames are shown at 8 frames per second.
(MOV)
