# Supplementary figures and images for: HAMLET Binding to α-Actinin Facilitates Tumor Cell Detachment
Source: PLoS One. 2011 Mar 8;6(3):e17179. doi: 10.1371/journal.pone.0017179 (PMC3050841; doi:10.1371/journal.pone.0017179)

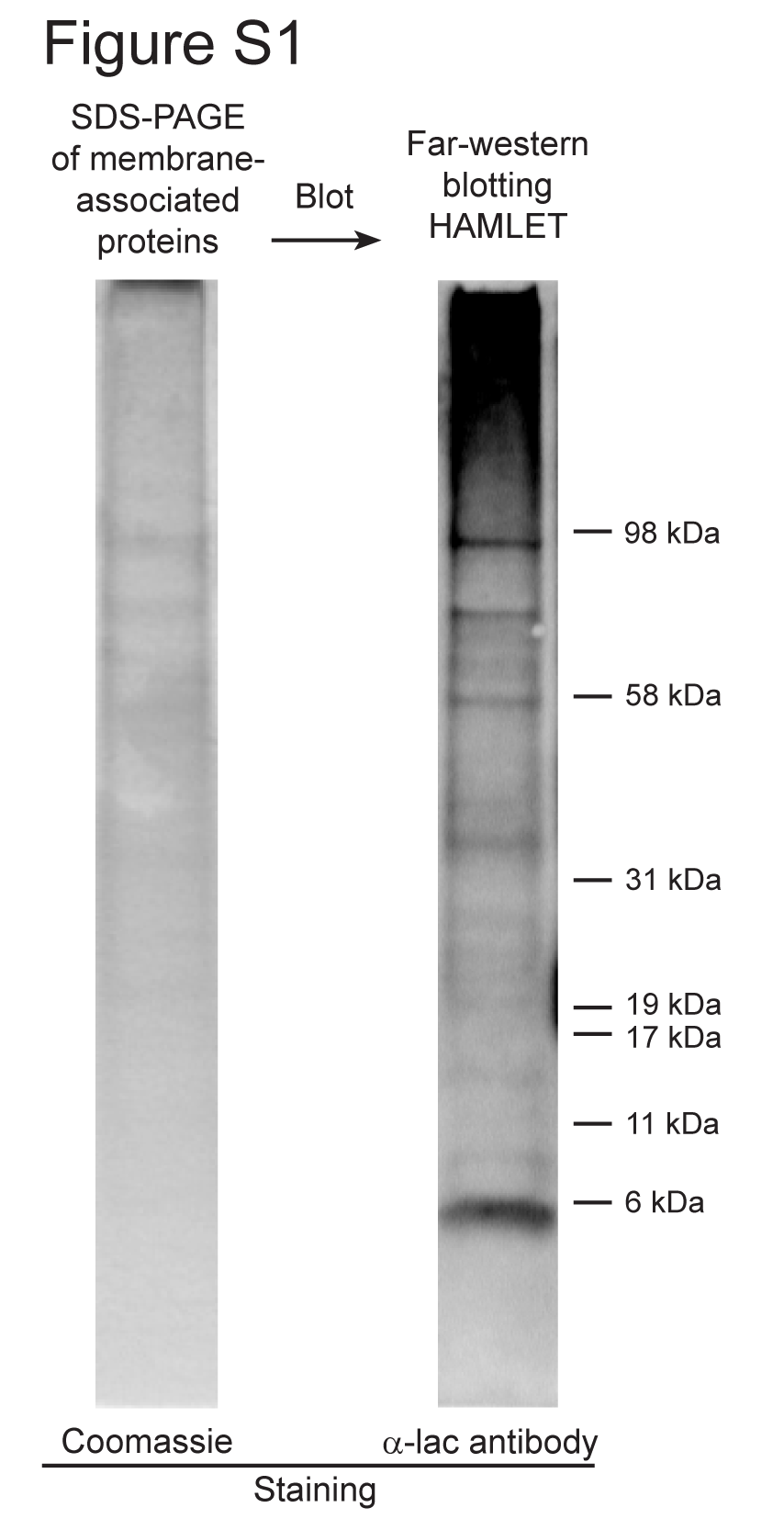

Supplement: Figure S1 — HAMLET binds to α-actinin-4. HAMLET was shown to bind to a 96 kDa protein in the membrane fraction of carcinoma cell extracts in a Far-Western blot with HAMLET. Bound HAMLET was detected using anti-α-lactalbumin antibodies. The 96 kDa band was identified as α-actinin-4 by mass spectrometry (Profound analysis). (TIF) [file pone.0017179.s001.tif]

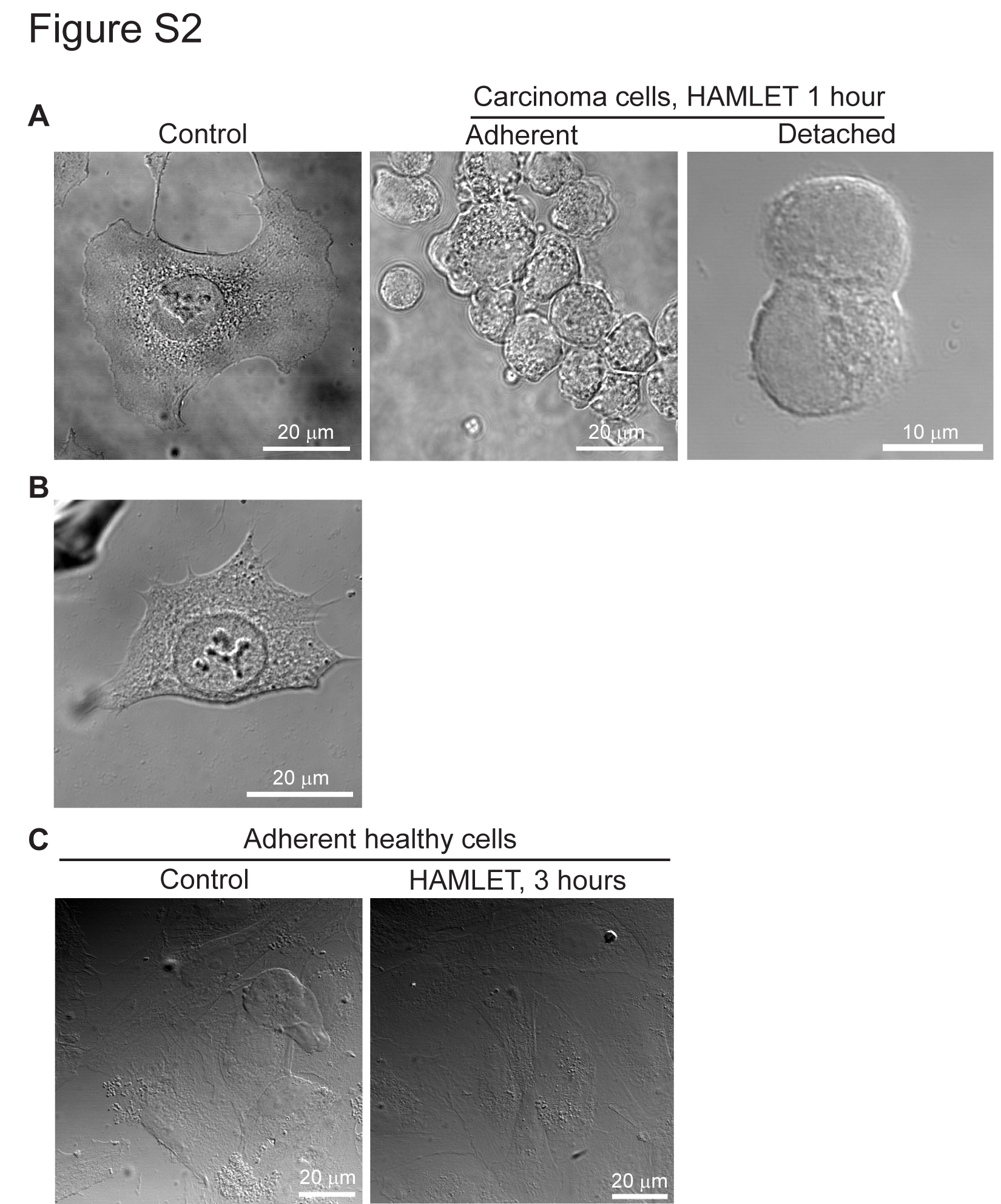

Supplement: Figure S2 — Light images. Light images corresponding to Figure 2. Tumor cells underwent morphological changes after HAMLET treatment while normal differentiated cells remained adherent with a flattened and distended morphology. (TIF) [file pone.0017179.s002.tif]

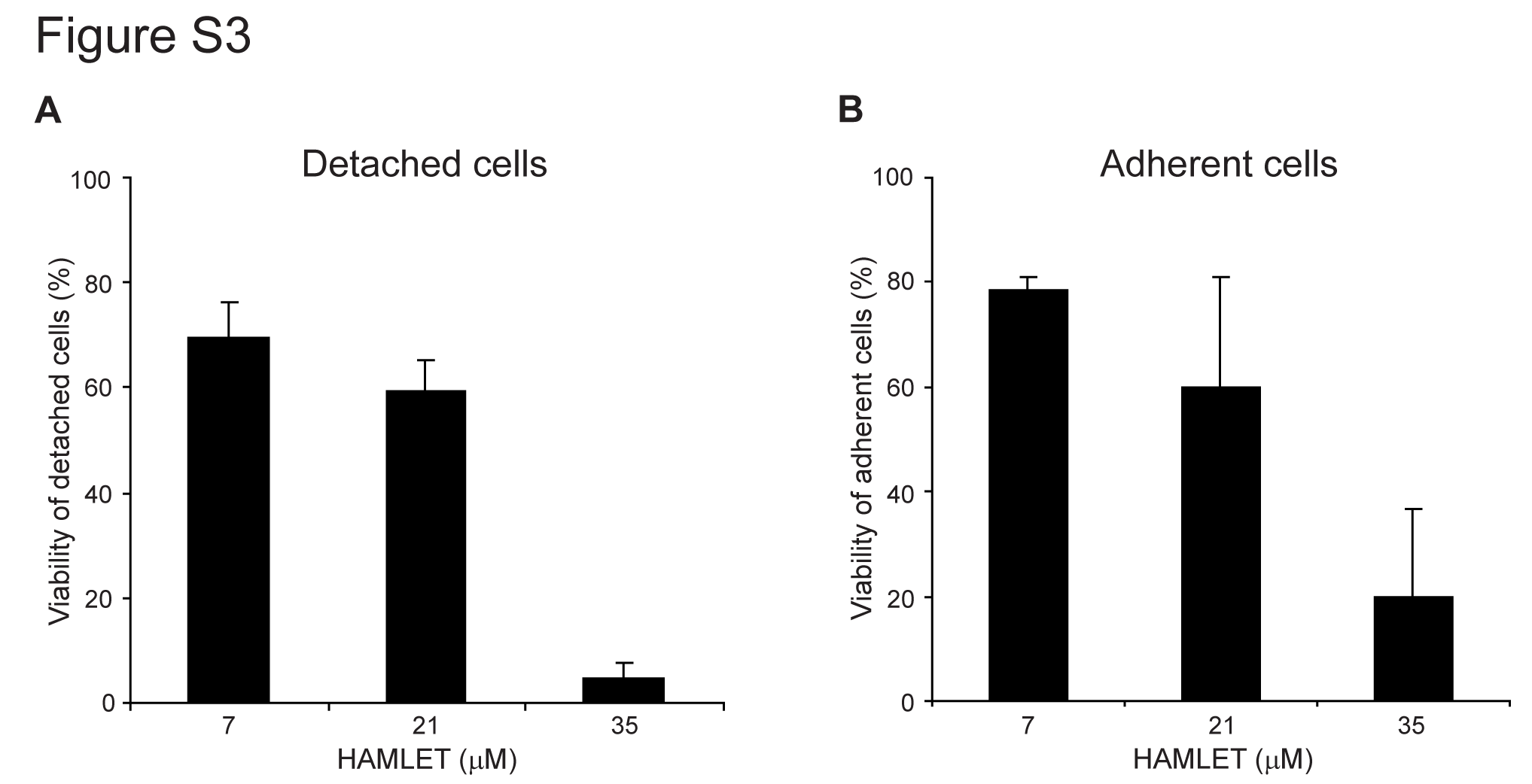

Supplement: Figure S3 — Cell viability in response to HAMLET. A) Adherent cells were treated with HAMLET and the cell viability of detached cells was quantified by trypan blue exclusion. After 3 hours of HAMLET treatment, cells treated with 7 and 21 µM of HAMLET that had detached showed 70 and 59% viability while the cells treated with 35 µM of HAMLET showed 5% viability (means + SEMs of 3 experiments). B) The cell viability of the remaining adherent cells was also quantified by trypan blue exclusion (means + SEMs of 2 experiments). (TIF) [file pone.0017179.s003.tif]

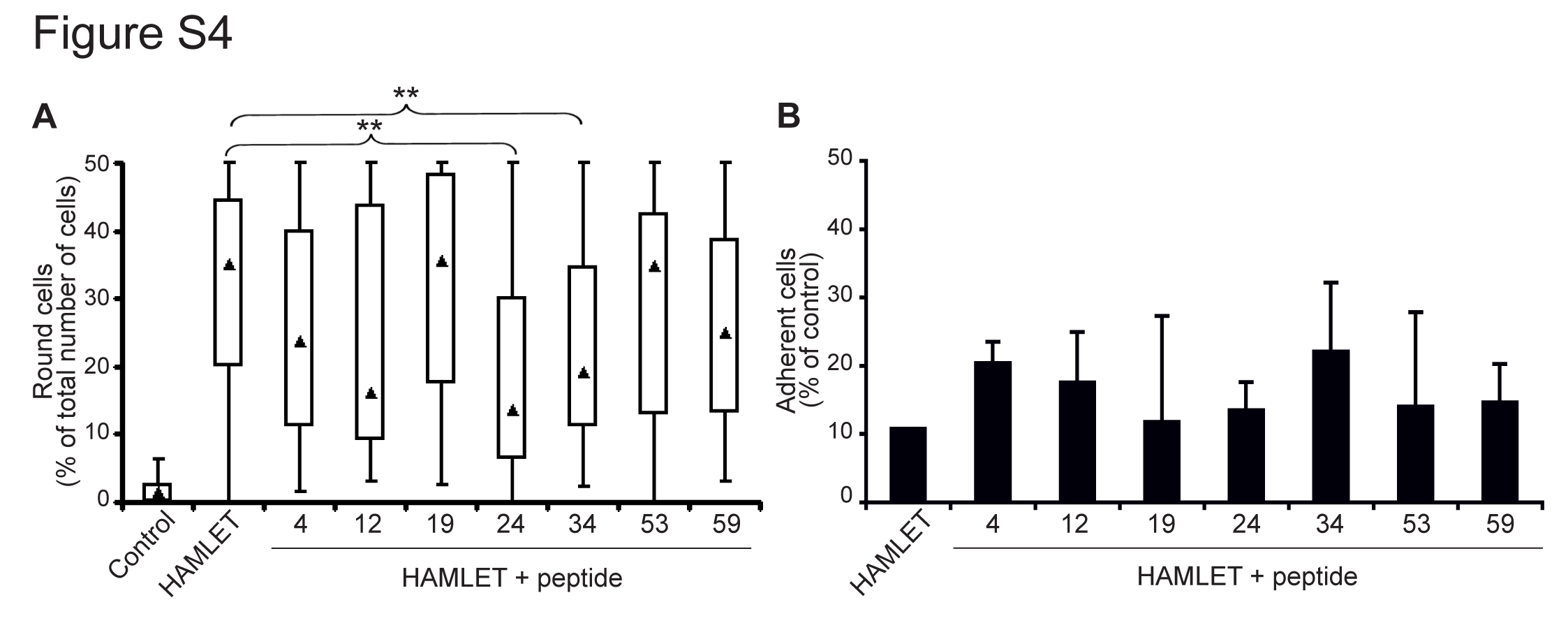

Supplement: Figure S4 — Inhibition of cell detachment by α-actinin-4 peptides. A, B) HAMLET was pre-incubated with the ten α-actinin-4 peptides that bound to HAMLET in the peptide-binding assay. The pre-incubated mixture or HAMLET alone was added to carcinoma cells grown on glass slides and the morphology (A) and detachment (B) were analyzed by light microscopy. Peptides 12 and 34 reduced the rounding up and the detachment of the carcinoma cells. Peptides 4 and 24 also inhibited cells from rounding up but the effects might be non-specific due to aggregate formation of HAMLET and peptide (A: 4 experiments; B: means + SEMs of 4 experiments; ** p<0.01). (TIF) [file pone.0017179.s004.tif]

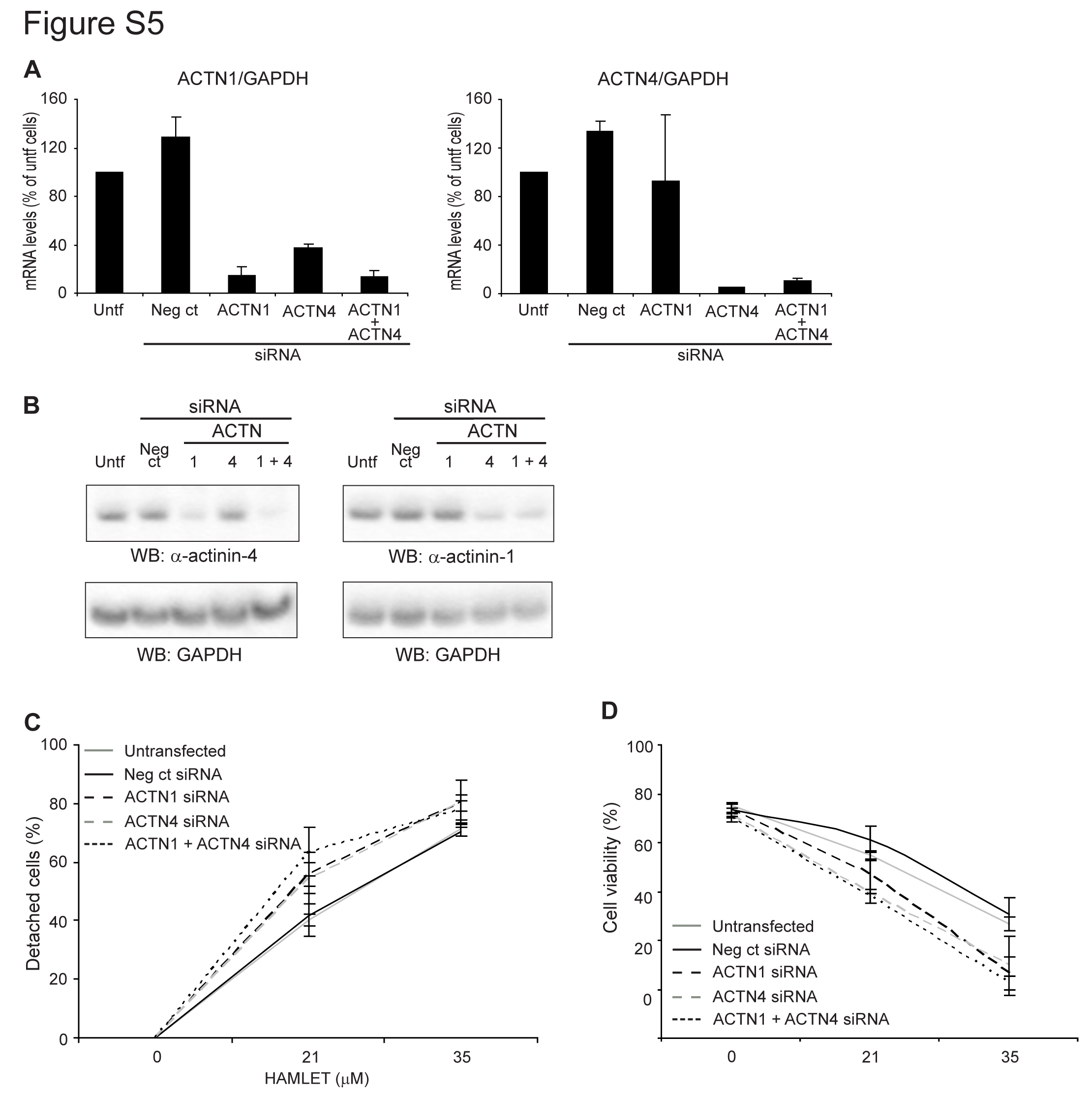

Supplement: Figure S5 — Altered α-actinin expression modifies cell detachment. A, B) α-Actinin siRNA knockdown was quantified by RT-PCR (A, means + SEMs of 3 experiments) and Western blot (B). C) The hexosaminidase activity assay was used to examine cell detachment after siRNA-treatment. In response to HAMLET tumor cell detachment was increased in cells with low α-actinin levels compared to cells transfected with negative control-siRNA or untransfected cells (means ± SEMs of 3 experiments). Cells transfected with both α-actinin-4- and α-actinin-1-specific siRNA showed a more pronounced detachment in response to HAMLET than cells treated with either siRNA alone. D) By Trypan blue exclusion, tumor cell death was increased in adherent cells with low α-actinin levels (means ± SEMs of 3 experiments). Cells transfected with both α-actinin-4- and α-actinin-1-specific siRNA showed a more pronounced cell death in response to HAMLET than cells treated with either siRNA alone. (TIF) [file pone.0017179.s005.tif]

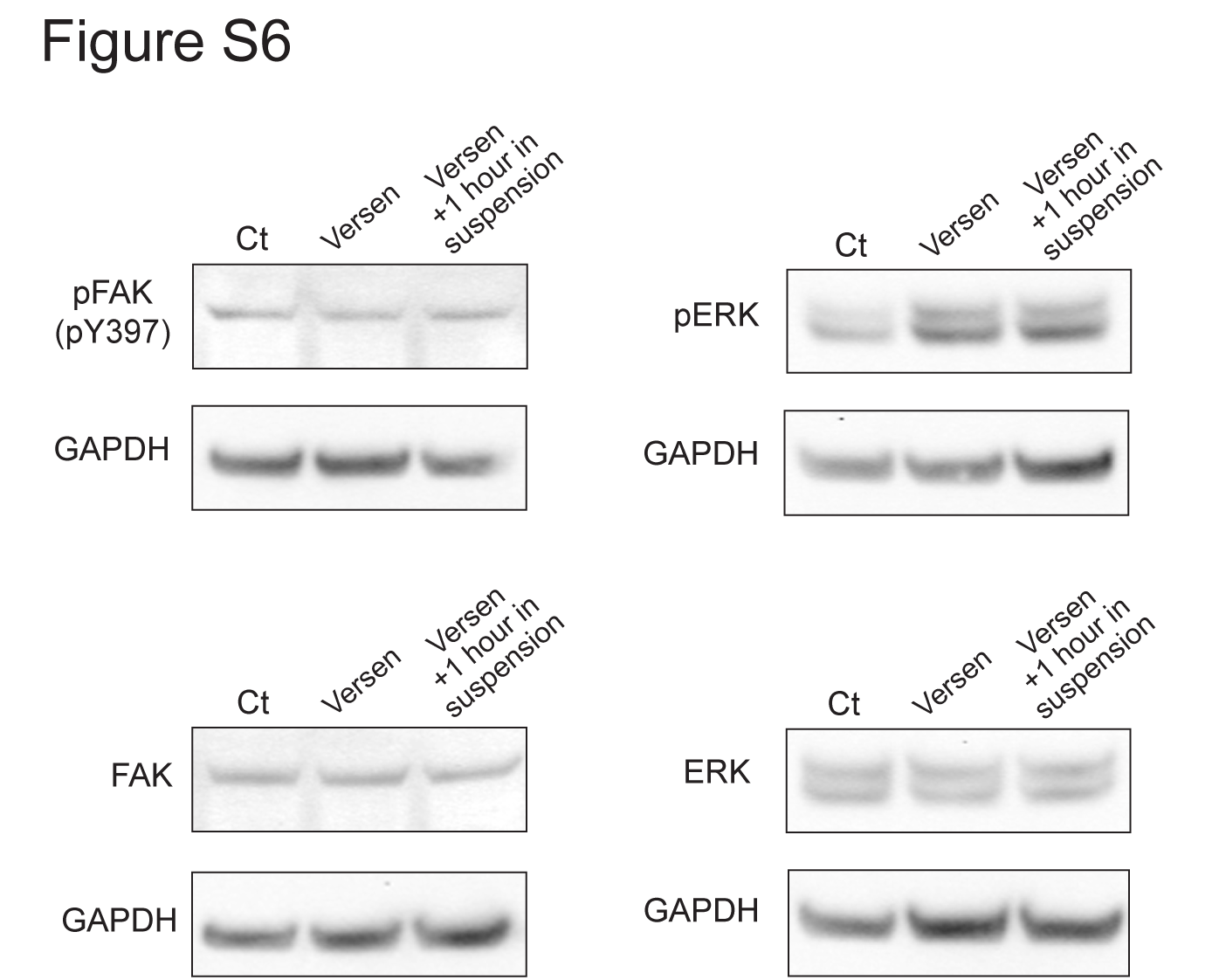

Supplement: Figure S6 — Detachment and FAK/ERK phosphorylation. A) Cells detached by EDTA treatment showed a slight reduction in FAK phosphorylation as shown by western blots using FAK and phospho-FAK (pTyr397) antibodies. GAPDH was used as loading control. B) Cells detached by EDTA treatment showed a slight increase in ERK phosphorylation as shown by western blots using ERK1/2 and phospho-ERK1/2 antibodies. GAPDH was used as loading control. (TIF) [file pone.0017179.s006.tif]

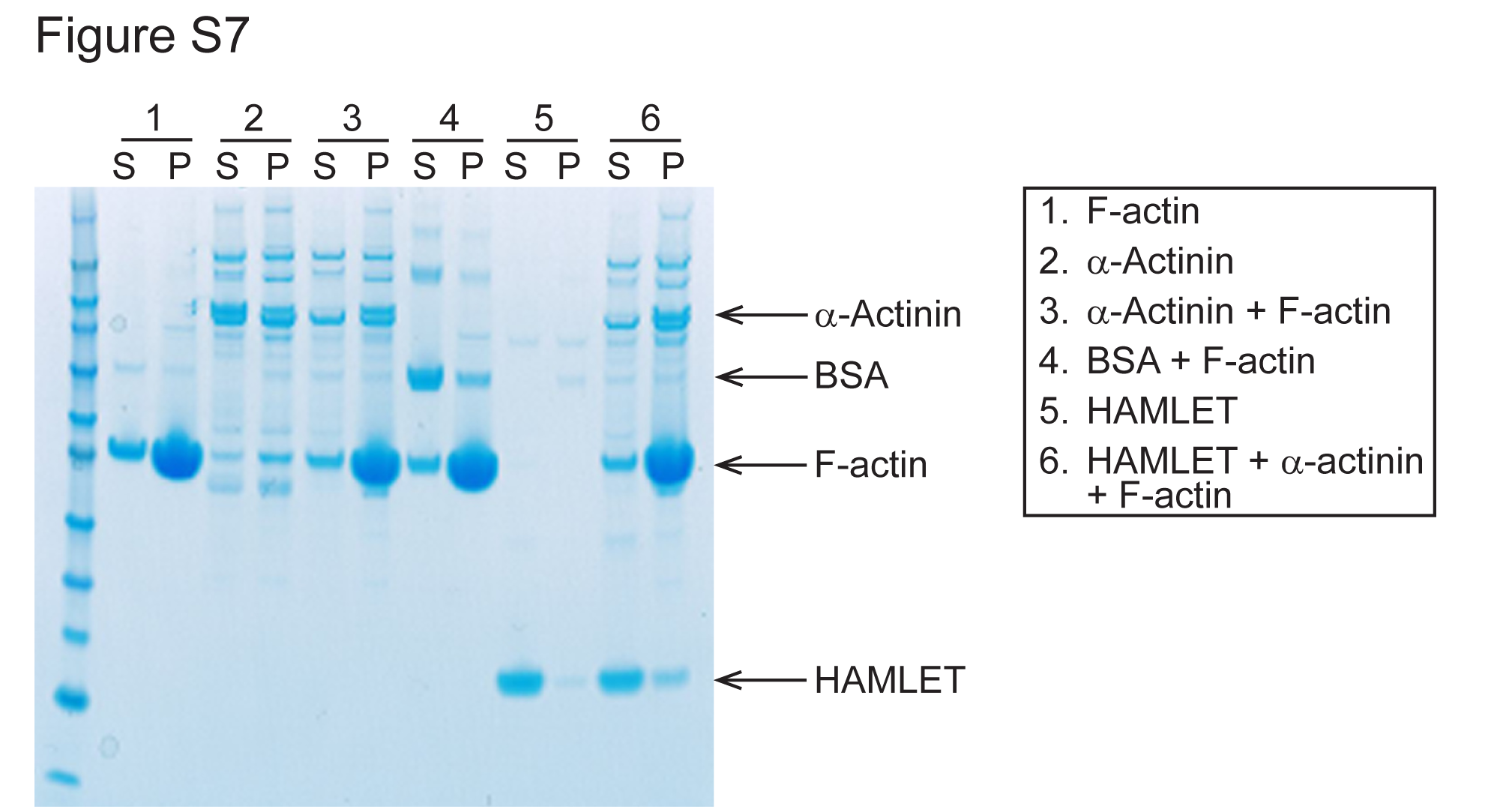

Supplement: Figure S7 — Actin-binding spin down assay. An actin-binding spin down assay was performed to examine if HAMLET disrupts the interaction of α-actinin with F-actin. After ultracentrifugation, F-actin forms a pellet (P) and actin-binding proteins are also detected in these fractions. Proteins that do not interact with actin remain in the supernatant (S). (TIF) [file pone.0017179.s007.tif]
