# Supplementary material for: HAMLET Binding to α-Actinin Facilitates Tumor Cell Detachment
Source: PLoS One. 2011 Mar 8;6(3):e17179. doi: 10.1371/journal.pone.0017179 (PMC3050841; doi:10.1371/journal.pone.0017179)
Supplement: Table S1 — Identification of α-actinin-4 by mass spectrometry. (DOC) [file pone.0017179.s008.doc]

**Table S1**. Identification of α-actinin-4 by mass spectrometry.

| Measured mass | Computed mass | Residues | Amino acid sequence |
| --- | --- | --- | --- |
| 863.473 | 863.475 | 115-122 | ALDFIASK |
| 1028.549 | 1028.529 | 312-319 | TIPWLEDR |
| 1173.642 | 1173.596 | 167-175 | EGLLLWCQR |
| 1214.704 | 1214.665 | 301-310 | LASDLLEWIR |
| 1385.818 | 1385.766 | 734-745 | VGWEQLLTTIAR |
| 1391.713 | 1391.640 | 55-65 | TFTAWCNSHLR |
| 1420.753 | 1420.698 | 396-406 | GYEEWLLNEIR |
| 1428.811 | 1428.756 | 746-757 | TINEVENQILTR |
| 1480.857 | 1480.803 | 312-323 | TIPWLEDRVPQK |
| 1519.790 | 1519.735 | 55-66 | TFTAWCNSHLRK |
| 1519.790 | 1519.735 | 54-65 | KTFTAWCNSHLR |
| 1560.765 | 1560.723 | 870-882 | ELPPDQAEYCIAR |
| 1607.842 | 1607.797 | 351-363 | CQLEINFNTLQTK |
| 1674.930 | 1674.893 | 854-868 | VLAGDKNFITAEELR |
| 1757.878 | 1757.815 | 498-512 | ICDQWDALGSLTHSR |
| 1772.955 | 1772.920 | 653-668 | QFASQANVVGPWIQTK |
| 3323.729 | 3323.739 | 696-723 | SIVDYKPNLDLLEQQHQLIQEALIFDNK |
